# Catch-Up of Routine School-Based Immunizations Since the COVID-19 Pandemic: 4-Year Observational Cohort Study

**DOI:** 10.2196/79669

**Published:** 2025-09-23

**Authors:** Kelvin Nartey, Laura Reifferscheid, Marilou Kiely, Shannon E MacDonald, Canadian Immunization Research Network

**Affiliations:** 1 Faculty of Nursing, University of Alberta, Edmonton Clinic Health Academy, 11405-87 Avenue, Edmonton, AB, T6G 1C9, Canada, 1 780-248-1563; 2 Department of Social and Preventive Medicine, Faculty of Medicine, Université Laval, Quebec City, QC, Canada

**Keywords:** vaccination, measles-mumps-rubella, human papillomavirus, hepatitis B, meningococcal conjugate ACYW-135, Tdap

## Abstract

**Background:**

School-based immunization programs (SBIPs) have been widely used to efficiently deliver vaccines to children and adolescents, achieving higher and more equitable coverage than community-based approaches, though implementation varies across regions. However, the COVID-19 pandemic disrupted SBIPs, leading to significant declines in vaccine coverage and highlighting the need for long-term assessments to determine the effectiveness of recovery efforts, especially as current research has largely overlooked SBIPs or focused narrowly on select vaccines.

**Objective:**

This study aimed to assess the impact of the COVID-19 pandemic and ongoing catch-up immunization efforts on vaccine coverage and administration volume among school-aged children in the Canadian province of Alberta. We also sought to determine the magnitude of any remaining vaccine deficits and estimate the duration of additional catch-up efforts required to address those deficits.

**Methods:**

In this retrospective cohort study, we used population-wide administrative health data to calculate monthly cumulative vaccine coverage among Grade 1, 6, and 9 cohorts, comparing four pandemic cohorts (2019‐2020, 2020‐2021, 2021‐2022, and 2022‐2023 grade-years) to a prepandemic cohort for each grade. We also calculated vaccine administration rates as monthly count of vaccines delivered, stratified by routine (ie, received during the school year as scheduled) and catch-up (ie, received after the regularly scheduled school year) doses. For each vaccine, we calculated vaccine coverage deficits remaining at the end of the study period and estimated time to clear those deficits based on observed catch-up uptake rates.

**Results:**

All Grade 6 pandemic cohorts had lower human papillomavirus (HPV) and hepatitis B vaccine coverage at the end of the school year (2.6‐60 percentage points lower and 1.2‐41.7 percentage points lower, respectively), though coverage had increased to near prepandemic levels after approximately 3 years of follow-up. Coverage among the Grade 1 and 9 cohorts remained below prepandemic levels at study end; the largest deficits were noted in the 2022‐2023 Grade 1 cohort (7.87 percentage points lower for measles-containing vaccines, 4.64 percentage points lower for pertussis-containing vaccines) and the 2020‐2021 cohort for Grade 9 (14.0 percentage points lower for meningococcal conjugate ACYW-135 vaccine, 12.9 percentage points lower for pertussis-containing vaccines). The additional catch-up time required to reach prepandemic coverage levels for all cohorts was estimated to be less than 1 year for Grade 6 cohorts, 5.6 years for Grade 1 cohorts, and 4.8 years for Grade 9 cohorts.

**Conclusions:**

Partial recovery of vaccine coverage was found for cohorts impacted by interruptions to school-based programming during the COVID-19 pandemic. Further efforts to alleviate coverage deficits among Grade 1 cohorts may be addressed in routine Grade 6 programs, while targeted efforts in postsecondary institutions may be required to address delays in Grade 9 immunizations.

## Introduction

School settings have been used in varying capacities around the world to administer vaccines to children and adolescents. Worldwide, the first grade is most frequently targeted for school-based immunization programs (SBIPs), though delivery to preadolescents and adolescents is increasing [1]. SBIPs are attractive, largely due to the ability to allow rapid delivery to a large portion of the target population [2], and have been shown to achieve higher and more equitable vaccine coverage than community-based delivery alone [1,3,4]. SBIPs have been used to support vaccine delivery across Canada for decades, though there is regional variation in the grades and vaccines offered, as well as how these settings are used for catch-up immunization and implementation of mandatory immunization policies [5].

Despite previous successes, school closures and social distancing measures implemented during the COVID-19 pandemic resulted in significant decreases in vaccine coverage among those routinely immunized through SBIPs [6]. These critical drops in vaccine coverage raised concerns about possible resurgences of vaccine-preventable diseases [7] and left immunization programs with the added challenge of identifying and vaccinating those who missed the recommended doses. In addition, increased parental vaccine hesitancy in some areas of the world [8] has led to concerns that decreases in vaccine coverage may persist or become more pronounced [9].

Research exploring pandemic-related disruptions to routine childhood immunization programs has focused mainly on children aged 5 years and younger [10,11] or adolescent coverage outside of SBIPs [12-15], with minimal attention paid to SBIPs to date [16]. Those evaluating coverage among children eligible for immunization through SBIPs have limited their analysis to select vaccines (human papillomavirus [HPV] vaccine only [17]; HPV and meningococcal conjugate ACYW-135 [MenC-ACYW] only [6]), providing a limited picture of the full extent and impact of catch-up activities. In addition, current evidence points to immunization deficits that have persisted past the initial months of pandemic-related health care and school disruptions [14,15,18]. Assessment of vaccine coverage over the longer term is needed to determine whether recovery efforts have been effective or additional strategies are needed.

The aim of this study was to assess the impact of the COVID-19 pandemic and ongoing catch-up immunization efforts on vaccine coverage and administration volume among school-aged children in the Canadian province of Alberta, using more than four years of observation since the pandemic. We also sought to determine the magnitude of any remaining vaccine deficits and estimate the duration of additional catch-up efforts required to address those deficits.

## Methods

### Study Design and Setting

This retrospective observational cohort study was conducted in Alberta, which has a population of 4.8 million people. Almost 99% of Albertans are registered with the universal, publicly funded Alberta Health Care Insurance Plan (AHCIP), which provides access to all vaccines included in the provincial routine immunization schedule free of charge. With the exception of travel and seasonal vaccines (for example, influenza and COVID-19), all vaccines for school-aged children are provided exclusively by public health nurses through community-based public health centers and SBIPs, which includes screening and vaccinating children in Grade 1, Grade 6, and Grade 9. The Grade 1 program acts as a catch-up opportunity for those who missed preschool immunizations, while the Grades 6 and 9 programs are the main touchpoints for offering scheduled adolescent vaccines [19]. While schools are used to offer and provide vaccines, no vaccines are mandatory for school attendance in Alberta. The province’s routine immunization schedule is provided in Multimedia Appendix 1. This study is reported in accordance with the STrengthening the Reporting of OBservational studies in Epidemiology (STROBE) cohort reporting guidelines [20].

### Data Sources and Population

The data set was created by linking the Provincial School Immunization Record (PSIR), which contains individual-level records of student enrollment for each year, including grade, age, and sex, with the Immunization and Adverse Reaction to Immunization (Imm/ARI) repository, which includes a record of all publicly-funded vaccines provided in the province. We excluded those who lived in the city of Lloydminster (as vaccines are delivered by a neighboring province), and those who died or left the province before the study end date. To focus on institutions with SBIPs, we excluded schools that offer online or distance education only, as well as postsecondary, continuing education, summer, or evening or weekend schools. Schools on First Nations reserves were also excluded, as immunization data is not consistently submitted to the Imm/ARI repository. To align with the standard ages of assessment in the province, we also restricted cohorts based on individual age as of September 01 of the school year, including only those 5‐7 years of age for Grade 1, 9‐11 years of age for Grade 5, 10‐12 years of age for Grade 6, and 13‐15 years of age for Grade 9.

We examined 5 annual cohorts for each grade targeted for SBIPs, including 4 pandemic cohorts (2019‐2020, 2020‐2021, 2021‐2022, and 2022‐2023) and 1 prepandemic cohort. For Grades 1 and 9, the prepandemic cohort was defined as the cohort eligible for immunization during the 2018‐2019 school year. For Grade 6, we used the 2017‐2018 Grade 5 group as the prepandemic cohort, as the target grade for vaccine delivery changed from Grade 5 to Grade 6 in 2018‐2019, resulting in no SBIP for Grade 5 or 6 in 2018‐2019.

For each grade, we focused on the vaccines routinely delivered through SBIPs, including a second dose of measles-mumps-rubella (MMR) or measles-mumps-rubella—varicella (MMRV), and fifth dose of diphtheria, tetanus, acellular pertussis, polio (DTap-IPV) in Grade 1; the 2-dose HPV and hepatitis B series in Grade 6; and, the single dose of MenC-ACYW and tetanus, diphtheria, and acellular pertussis (Tdap) series in Grade 9. We included all pertussis-containing vaccines in the DTaP-IPV (Grade 1) and Tdap (Grade 9) analyses.

### Data Analysis

For each vaccine, stratified by grade-year cohort, we calculated cumulative vaccine coverage, that is, the proportion of the grade-year cohort who had received the vaccine dose of interest, calculated for each month of observation. We determined cumulative coverage for each cohort from the end of their respective school year (eg, July 2020 for the 2019‐2020 cohorts) to the end of the study period (July 2024), aiming to capture the impact of both the intensive and routine catch-up activities. As the Grade 1 immunization program is a routine catch-up opportunity (ie, children would have had the opportunity to receive the vaccines of interest before entering their Grade 1 year), we included monthly cumulative coverage from the start of each cohort’s Grade 1 year (eg, September 2019 for the 2019‐2020 cohorts). For the Grade 6 HPV and hepatitis B series, we calculated both complete (2 dose) and 1+ dose coverage, given recent recommendations that 1 dose of HPV vaccine may confer immunity. During the prepandemic (2017‐2018) school year, a 3-dose program for both HPV and hepatitis B was in place, which subsequently switched to a 2-dose program; thus, complete coverage was defined as receipt of 3 doses for the 2017‐2018 cohort, and at least 2 doses for all subsequent cohorts. To estimate pandemic impacts, we calculated three measures of comparison for each vaccine of interest, using the prepandemic cohort as the counterfactual for pandemic cohorts: (1) cumulative coverage difference, (2) vaccine administration volume, and (3) cumulative immunization deficits.

To determine cumulative coverage differences, cumulative monthly coverage for each pandemic cohort was compared to the analogous month for the prepandemic cohort. For example, the July 2021 coverage for the 2019‐2020 pandemic cohort represents coverage at 12 months from end of grade year and thus was compared to the coverage for the prepandemic cohort at 12 months from the end of grade year. We calculated 95% CI for binomial proportions, for both coverage and difference in coverage.

Vaccine administration, that is, a count of the number of vaccine doses delivered, was assessed for each grade-year cohort for each month of observation, collapsed into “school year” (from September to June) and “summer” (July and August) for each study year. We calculated administration for 1 vaccine for each grade: for Grade 6 and 9, we chose the vaccine with the highest administration volume (HPV and MenC-ACYW, respectively); for Grade 1, we chose to assess MMR or MMRV administration, given currently ongoing measles outbreaks globally. Administration counts were stratified by “routine” (ie, received during the school year as scheduled) and “catch-up” (ie, received after the regularly scheduled school year) immunization. We used dose count rather than calculating a delivery rate, which would correct for differences in cohort sizes, as we were interested in the magnitude and persistence of changes to vaccine administration volume associated with catch-up efforts.

We calculated the cumulative deficits for each vaccine at the end of the study period by comparing the observed number of individuals vaccinated for all pandemic cohorts to an expected number of individuals, estimated based on vaccine coverage for the prepandemic cohort at the end of the study period. For each pandemic cohort, the expected number of individuals vaccinated at the end of the study period was calculated by multiplying the observed cohort vaccine coverage at the end of the study period by the number of individuals in the pandemic cohort. We then summed the expected numbers of individuals vaccinated for all pandemic cohorts and calculated the difference from the observed number vaccinated at the end of the study period. Finally, we compared this calculated difference to observed maximum and average annual catch-up vaccine uptake, that is, number of individuals vaccinated [21], to estimate time to clear any accumulated deficits in coverage. All analyses were completed using SAS (version 9.4; SAS Institute Inc), and Microsoft Excel (version 1808).

### Ethical Considerations

This study received approval from the Health Research Ethics Board (HREB) at the University of Alberta (Pro00134011). Informed consent to participate was waived by the HREB. All study data were deidentified.

## Results

A total of 799,961 children were included in our study cohorts; 270,603 were in the Grade 1 cohorts, 273,163 in Grade 6, and 256,195 in Grade 9. The total number in each grade-year cohort is provided in Multimedia Appendix 1.

### Cumulative Coverage Difference

**Figure 1.**
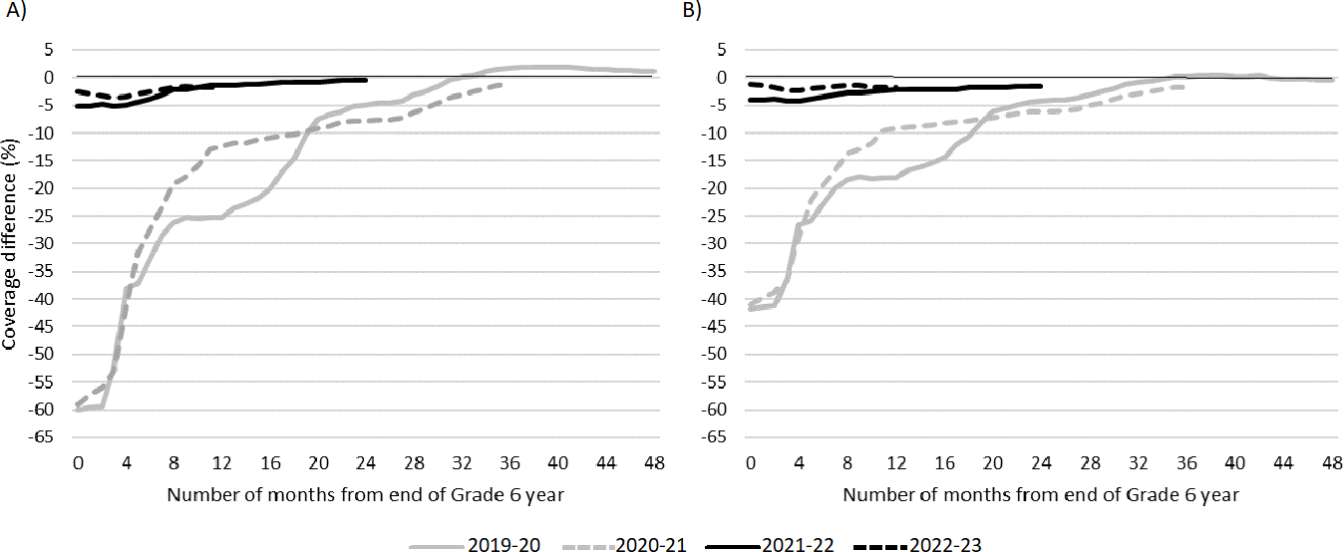
Differences in monthly complete (2 dose) coverage compared to prepandemic school year (2017‐2018) for each Grade 6 cohort for: (A) human papillomavirus (HPV) and (B) hepatitis B. Differences were calculated by comparing the cumulative monthly coverage for each pandemic cohort to the cumulative coverage in the analogous month for the prepandemic cohort.

Cumulative vaccine coverage for the Grade 6 pandemic cohorts was lower than prepandemic levels at the end of each school year, although they reached at or near prepandemic coverage by the end of the observation period (see Figure 1). The 2019‐2020 and 2020‐2021 cohorts finished their school years with the largest deficits: 60 and 59 percentage points lower for HPV (see Figure 1A and Table S2 in Multimedia Appendix 1); and 41.7 and 41.0 percentage points lower for hepatitis B (see Figure 1B and Table S3 in Multimedia Appendix 1). For the 2019‐2020 cohort, coverage levels were at or above prepandemic levels by February (HPV) and May (hepatitis B) of 2023. Another cohort, the 2021‐2022 cohort, reached prepandemic levels for HPV by the end of the study period. For all other cohorts, coverage levels were less than 2 percentage points lower than prepandemic levels by the end of the observation period for both vaccines. For comparison, coverage for the prepandemic cohort ranged from 65.6% (end of Grade 6 year) to 75.4% (at 48 months of follow-up) for HPV (see Table S2 in Multimedia Appendix 1), and 73.7% (end of Grade 6 year) to 83% (at 48 months of follow-up) for hepatitis B (see Table S3 in Multimedia Appendix 1). HPV vaccine coverage remained below hepatitis B coverage levels throughout the study period, for all cohorts. Cumulative 1+ dose coverage for the most impacted cohort (2020‐21) was approximately 15 percentage points lower than prepandemic levels at the end of the school year for both vaccines, recovering to within 2 percentage points of prepandemic levels within 31 (HPV) and 36 (hepatitis B) months of observation (see Figure S1 in Multimedia Appendix 1).

**Figure 2.**
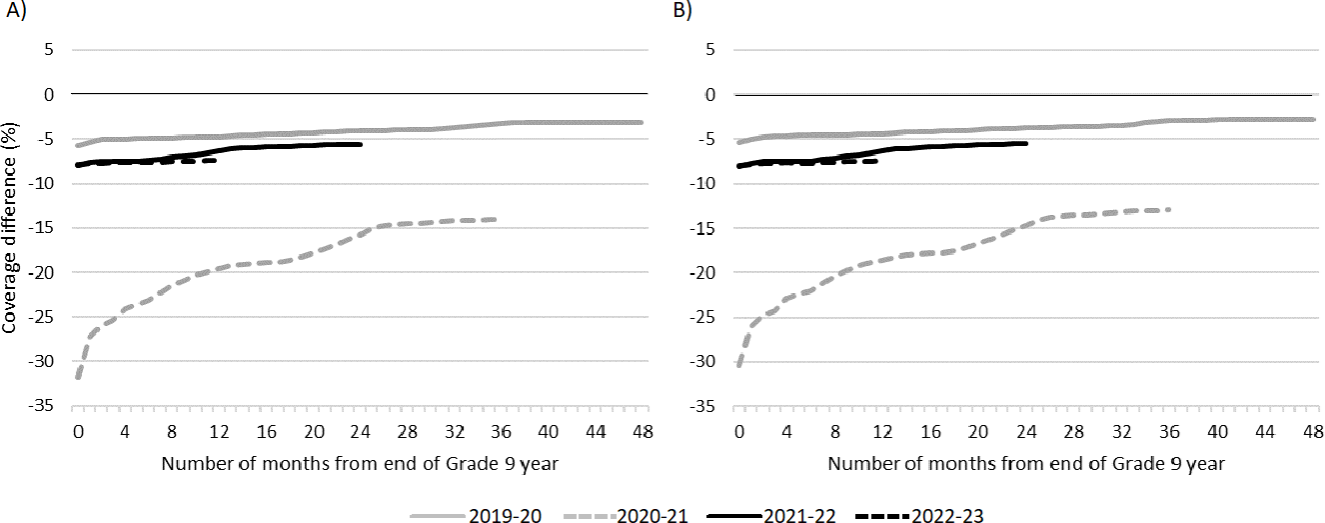
Differences in monthly complete coverage compared to prepandemic school year (2018‐19) for each Grade 9 cohort for: (A) Meningococcal conjugate ACYW-135 (MenC-ACYW; 1 dose), and (B) Tetanus, diphtheria, and acellular pertussis (Tdap; 1 dose). Differences were calculated by comparing the cumulative monthly coverage for each pandemic cohort to the cumulative coverage in the analogous month for the prepandemic cohort.

Cumulative coverage for all Grade 9 pandemic cohorts remained below prepandemic coverage throughout the study period (see Figure 2). The largest deficits in coverage were observed for the 2020‐2021 Grade 9 cohort, with coverage for both vaccines more than 30 percentage points lower than prepandemic at the end of the school year, with deficits persisting through to the end of observation: 14 percentage points (95% CI 13.5‐14.5) lower for MenC-ACYW coverage (see Figure 2A and Table S4 in Multimedia Appendix 1) and 12.9 percentage points (12.4‐13.4) lower for Tdap (see Figure 2B and Table S5 in Multimedia Appendix 1). Coverage for the prepandemic cohort ranged from 84.9% (at end of Grade 9 year) to 86.6% (after 48 months of follow-up) for MenC-ACYW (see Table S4 in Multimedia Appendix 1), and from 85.6% (at end of Grade 9 year) to 88.2% (at 48 months of follow-up) for Tdap (see Table S5 in Multimedia Appendix 1).

**Figure 3.**
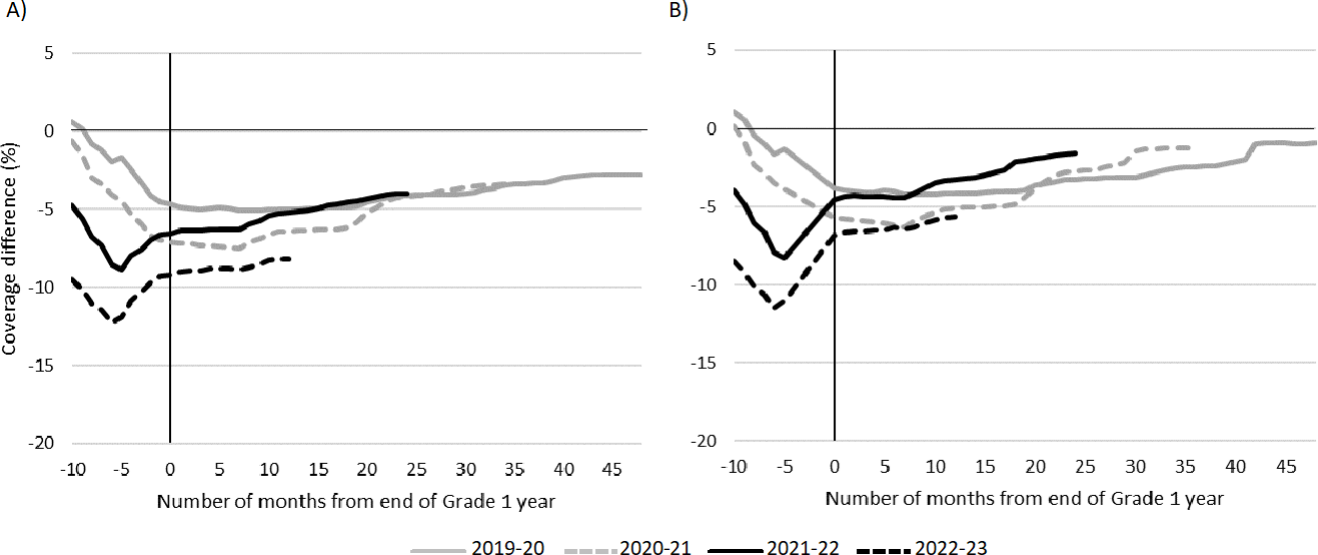
Differences in monthly complete coverage compared to prepandemic school year (2018‐19) for each Grade 1 cohort for: (A) Measles-mumps-rubella (MMR) or measles-mumps-rubella—varicella (MMRV; 2 doses), and (B) Diphtheria, tetanus, acellular pertussis, and polio (DTap-IPV; 5 doses). Differences were calculated by comparing the cumulative monthly coverage for each pandemic cohort to the cumulative coverage in the analogous month for the prepandemic cohort.

For all Grade 1 pandemic cohorts, cumulative coverage remained below prepandemic levels throughout the observation period, for both MMR or MMRV and DTaP-IPV (see Figure 3). For both vaccines, coverage at the start of the Grade 1 year was similar to prepandemic levels for the 2019‐2020 and 2020‐2021 cohorts, but significantly lower for the 2021‐2022 and 2022‐2023 cohorts. During the school year, coverage for the 2019‐2020 and 2020‐2021 cohorts continued to decline relative to prepandemic levels. In contrast, the 2021‐2022 and 2022‐2023 cohorts ended their Grade 1 years with coverage deficits similar to the start of the school year. For all cohorts, coverage deficits gradually decreased during the remaining period of observation. MMR or MMRV coverage for all cohorts remained significantly below prepandemic levels at the end of the study period, ranging from 3.06 percentage points lower (2.69‐3.44) for the 2019‐2020 cohort, to 7.87 percentage points lower (7.46‐8.28) for the 2022‐2023 cohort (see Figure 3A and Table S7 in Multimedia Appendix 1). Smaller deficits were observed for DTaP-IPV coverage, ranging from 0.5 percentage points lower (0.11‐0.92) in the 2019‐2020 cohort to 4.64 percentage points lower (4.20‐5.08) in the 2022‐2023 cohort (see Figure 3B and Table S6 in Multimedia Appendix 1). Coverage for the prepandemic cohort increased during the period of comparison: from 76.6% (76.3%‐77%; September 2018) to 90.5% (90.2%‐90.7%; July 2023) for MMR or MMRV (see Table S7 in Multimedia Appendix 1); from 74.3% (74%‐74.7%; September 2018) to 87.1% (86.8%‐87.4%; July 2023) for DTap-IPV (see Table S6 in Multimedia Appendix 1). Compared to DTaP-IPV, generally larger deficits in MMR or MMRV coverage were observed in all pandemic Grade 1 cohorts; MMR or MMRV coverage remained higher than DTaP-IPV in the 2019‐2020, 2020‐2021, and 2021‐2022 cohorts, but was lower in the 2022‐2023 cohort.

### Vaccine Administration

**Figure 4.**
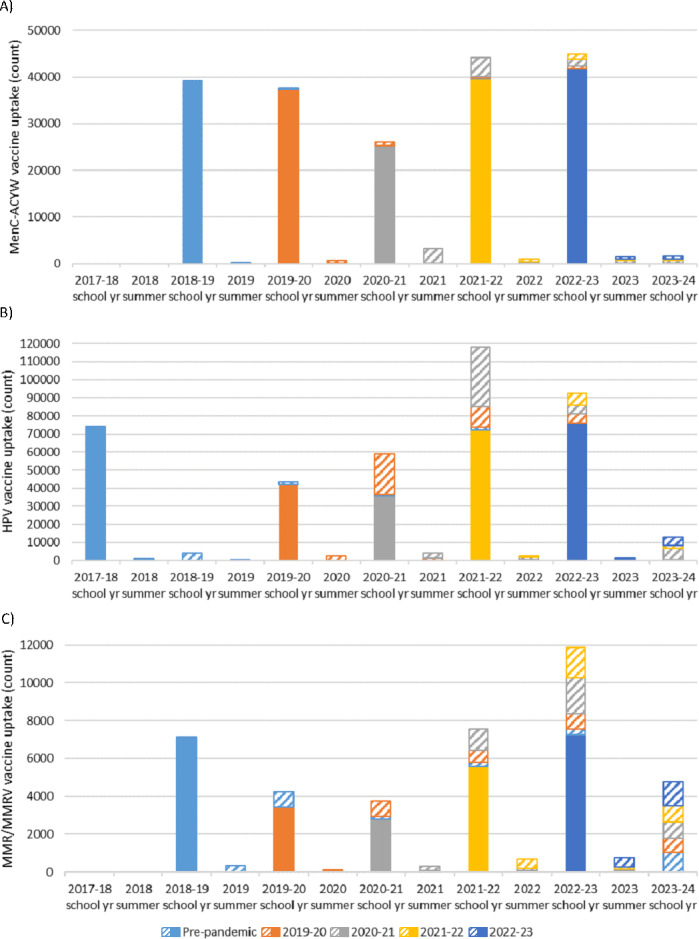
Vaccine administration volume for: (A) Meningococcal conjugate ACYW-135 (MenC-ACYW), **(B**) human papillomavirus (HPV), and (C) measles-mumps-rubella (MMR) or measles-mumps-rubella—varicella (MMRV) by grade-year cohort. Solid bars represent routine vaccine administration, and hatched bars represent catch-up vaccine administration.

Drops in vaccine administration (ie, count of the number of vaccine doses delivered) were observed during the 2019‐2020 and 2020‐2021 school years, with volume increasing to above prepandemic levels during the 2021‐2022 and 2022‐2023 school years (see Figure 4). For Grade 1, the largest volume of catch-up immunization occurred during the 2023-2024 year (2023 summer and 2023‐2024 school year [from July 2023 to June 2024], total of 5521 MMR or MMRV doses delivered). For both Grade 6 and Grade 9, the largest volume of catch-up immunization was completed during the 2021-2022 year (from July 2021 to June 2022), with totals of 50,290 (HPV) and 7780 (MenC-ACYW) doses delivered, respectively. Immunization of the prepandemic cohort continued throughout the study period for all vaccines, with vaccine delivery observed during each summer and school year included. While generally higher vaccine administration volumes were noted during the school years, we are unable to identify whether those doses were delivered through SBIPs or through other public health vaccination efforts.

### Cumulative Immunization Deficits

The cumulative deficits of unimmunized or incompletely immunized individuals for each vaccine, aggregated for all years, are presented in Table 1. The estimated time to clear these deficits is also presented, including the minimum time required (based on maximum effort observed in the previous years) and average time required (based on the average effort observed in the previous years). Deficits were highest for the Grade 1 and Grade 9 cohorts, with correspondingly greater time estimated to clear these deficits.

**Table 1.** Cumulative vaccine deficits among pandemic cohorts at the end of the study period (July 2024), relative to maximum vaccine coverage for prepandemic grade cohorts, and estimated time to clear deficit.

Grade and vaccine		Number of individuals vaccinated		Cumulative deficit	Estimated remaining catch-up time (years)	
		Expected^a^	Observed		Minimum^b^	Average^c^
Grade 1						
DTaP-IPV^d^		198,332	183,132	−15,200	3.1	5.6
MMR^e^ or MMRV^f^		199,198	184,409	−14,789	3.0	5.4
Grade 6						
HPV^g^		167,633	161,700	−5933	0.14	0.37
Hepatitis B		186,029	176,768	−9261	0.22	0.78
Grade 9						
MenC-ACYW^h^		180,871	164,436	−16,435	2.1	4.8
Tdap^i^		185,057	167,623	−17,434	2.2	4.7

a Estimated number of individuals vaccinated in pandemic cohorts to achieve coverage equivalent to maximum coverage observed in pre-pandemic cohort.

b Calculated using maximum annual catch-up uptake observed for each grade.

c Calculated using average annual catch-up uptake observed for each grade.

d DTaP-IPV: diphtheria, tetanus, acellular pertussis, and polio.

e MMR: measles-mumps-rubella.

f MMRV: measles-mumps-rubella—varicella.

g HPV: human papillomavirus.

h MenC-ACYW: meningococcal conjugate ACYW-135.

i Tdap: tetanus, diphtheria, and acellular pertussis.

## Discussion

### Principal Findings

We explored the cumulative impact of immunization program interruptions and subsequent catch-up efforts that have occurred since the COVID-19 pandemic on vaccine coverage and administration among school-aged children. Our results indicate that catch-up efforts resulted in significant recovery of vaccine coverage levels; however, important gaps remain. HPV and hepatitis B vaccine coverage levels had reached or were near prepandemic levels in all pandemic Grade 6 cohorts by the end of follow-up, though recovery took approximately 36 months for cohorts with the most significant relative deficits in coverage (2019‐20 and 2020‐21 grade-year). Our findings regarding persistent coverage deficits among Grade 1 and Grade 9 cohorts, more than 3 school years after pandemic-related school closures, are particularly concerning, given the recent elevation in pertussis [22] and measles [23] cases recorded in Canada. Both programs face unique challenges, with vaccine delivery to Grade 1 cohorts deprioritized early in the pandemic, and limited access to Grade 9 cohorts as they age out of routine SBIPs. While the routine SBIPs provide additional opportunities to catch up Grade 1 cohorts, efforts outside of secondary schools will be required to reach Grade 9 students who remain undervaccinated.

We found significant, though largely temporary, drops in HPV and hepatitis B vaccine coverage among Grade 6 cohorts. These results indicate that potential changes in immunization attitudes had little to no impact on uptake for these vaccines. This is particularly important to note for the HPV vaccine, which has historically been subject to large drops in uptake due to changes in public sentiment [24]. Similar to our findings, a largely temporary drop in HPV and hepatitis B vaccine coverage was observed in the province of Quebec, which also has a well-established SBIP [17]. However, that study observed a return to near prepandemic 1+ dose coverage levels after only one year of catch-up efforts for the most impacted cohort (2020‐2021 school year), after experiencing drops in coverage larger than reported here, highlighting the importance of contextualizing findings within regional strategies used for intensive catch-up programs. While results from Alberta indicate that catch-up efforts were successful at returning coverage to prepandemic levels, coverage of both HPV and hepatitis B remains significantly below national vaccine coverage goals of 90% [25].

We found catch-up immunization efforts for Grade 1 cohorts were delayed relative to the Grade 6 and 9 cohorts, confirming that primary vaccine series were prioritized in catch-up efforts [26]. Concerningly, the most recent (2022‐2023) Grade 1 cohort in our study showed the largest coverage deficit compared to prepandemic levels. This cohort would have been scheduled to receive their preschool vaccines during the initial stages of the pandemic, when access to immunization services was limited and infant vaccines were prioritized [27]. Other studies also noted relatively larger declines in immunization for this age group compared to infants [13,15,28,29]. While the Grade 1 SBIP coverage improvements for this cohort were similar to the SBIP in the prepandemic cohort, deficit levels from the preschool program remain. It will be important to understand whether the limited catch-up during the Grade 1 program is due to individual (eg, increased vaccine refusal, decreased school attendance) or programmatic (eg, staff capacity, school access) factors.

Our results indicate that catch-up efforts impacted coverage for all cohorts, across all vaccines, throughout the study period, demonstrating the importance of maximizing opportunities for immunization. While intensive catch-up in different grades was prioritized at different times, these efforts represent notable increases in vaccine delivery workload. In addition, these numbers do not capture unseen efforts related to mitigating potentially increased student stress [30], and heightened school collaboration required to implement catch-up immunization efforts. It is unclear whether it is feasible for providers to sustain this level of catch-up efforts for the estimated additional time required to address outstanding deficits. Finally, while ongoing catch-up efforts can simplify vaccine access and prompt vaccine uptake, they also have the potential to contribute to vaccine fatigue. Vaccine fatigue, described as individual “inertia or inaction towards vaccine information or instruction due to perceived burden and burnout” [31], has the potential to lead to ongoing decreases in vaccine uptake. More information on the impact of repeat offering is needed to determine whether those subsequently vaccinated simply missed previous opportunities, or their willingness to be vaccinated changed due to programmatic (eg, removing COVID-19 immunization from schools) or contextual (eg, infectious disease outbreaks) factors. While ongoing monitoring of vaccine-specific coverage deficits can help appropriately direct program efforts, optimizing the use of schools for catch-up programs will require careful consideration of the impact of these efforts on providers, children and their parents or caregivers, and schools.

### Strengths and Limitations

This study provides a comprehensive summary of how vaccine coverage among children targeted through routine SBIPs was impacted by the COVID-19 pandemic and subsequent catch-up immunization efforts. Rather than evaluating a 1-time measure of vaccine coverage at a set age, we followed each cohort after grade completion to study end (July 2024). As a result, total cumulative vaccine coverage for our prepandemic comparison cohorts reflects vaccine activity that occurred before (ie, routine) and during the pandemic (ie, catch-up). Thus, the observed catch-up vaccination for this cohort may also reflect pandemic-related decreases in vaccination activity, causing an underestimation of pandemic impacts by the end of our study period.

Our work benefited from a population-based vaccine repository with information on all vaccines provided in the province, as well as complete denominator data. However, it is possible that vaccines received outside of the province were not completely captured in the vaccine repository, which could lead to underestimations in coverage. We also did not capture prepandemic trends that may have inflated or deflated coverage differences noted for the pandemic cohorts. Our HPV and hepatitis B prepandemic comparison year reflected coverage for the 3-dose series offered to Grade 5 students, which we compared to cohorts exposed to the 2-dose series offered in Grade 6. The decrease in series doses could have contributed to lower coverage deficits noted in the pandemic cohorts. We were also unable to identify whether catch-up vaccines were delivered through school programs or through other efforts (eg, outreach programs and community clinic visits). Future work should consider the impact of catch-up efforts on inequities in vaccine coverage.

### Conclusions

We found coverage deficits that occurred during and after the COVID-19 pandemic persisted for at least 3 years after the pandemic-related school and health facility closures were initiated in March 2020. While we noted recovery to prepandemic vaccine coverage levels for vaccines scheduled in Grade 6, deficits remain in those targeted for delivery in Grades 1 and 9. Existing Grade 6 and 9 routine programs may provide additional opportunities to address immunization gaps among younger cohorts; strengthening these should be a priority. However, reaching those who missed Grade 9 vaccines will require targeted efforts in high school and post-secondary educational institutions. A better understanding of the approaches used for successful catch-up, in the context of routine delivery approaches, is required to ensure intensive and routine catch-up programs are optimized.

## Supplementary material

10.2196/79669 Multimedia Appendix 1 Supplementary figures and tables.

## Abbreviations

DTap-IPV: diphtheria, tetanus, acellular pertussis, and polio
HPV: human papillomavirus
MenC-ACYW: meningococcal conjugate ACYW-135
MMR: measles-mumps-rubella
MMRV: measles-mumps-rubella—varicella
SBIP: school-based immunization program
Tdap: tetanus, diphtheria, and acellular pertussis
