# Supplementary material for: Catch-Up of Routine School-Based Immunizations Since the COVID-19 Pandemic: 4-Year Observational Cohort Study
Source: JMIR Public Health Surveill. 2025 Sep 23;11:e79669. doi: 10.2196/79669 (PMC12456353; doi:10.2196/79669)
Supplement: Multimedia Appendix 1 [file publichealth-v11-e79669-s001.pdf]

Supplementary File

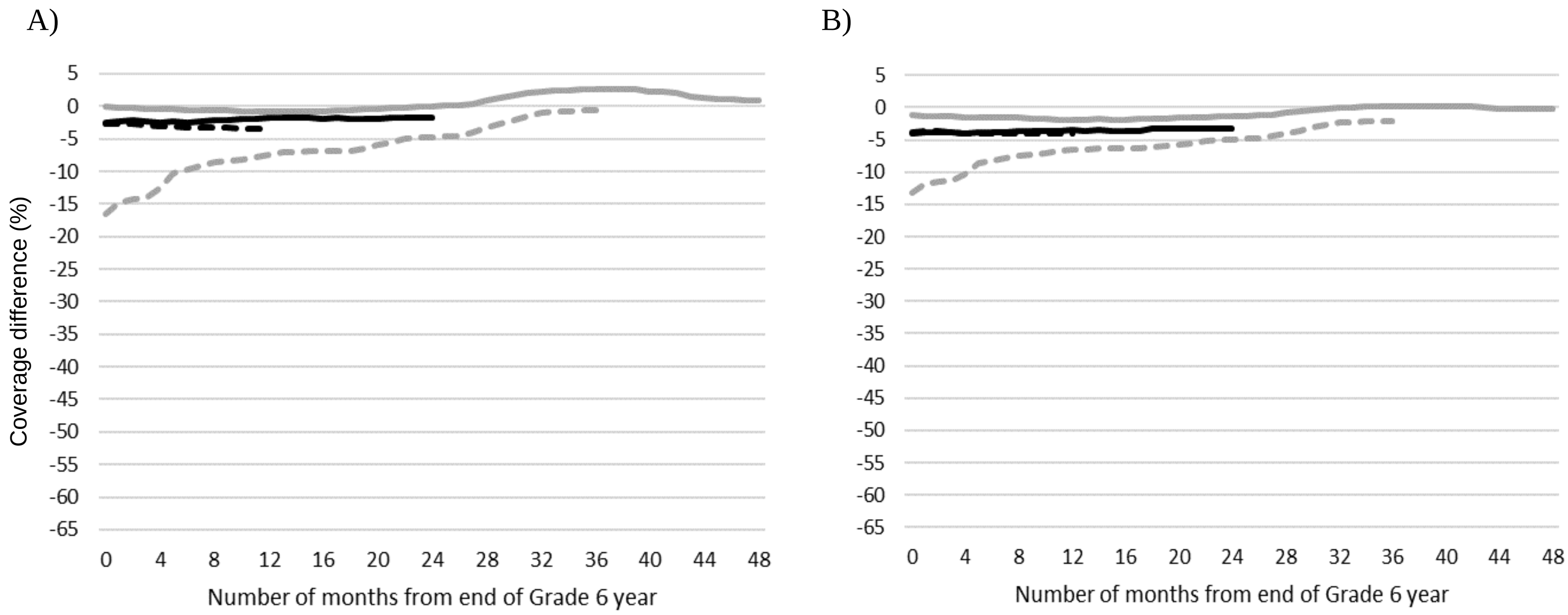

Figure S1. Differences in 1+ dose coverage compared to pre-pandemic school year (2017-18) for each Grade 6 cohort for: a) HPV; and, b) HB. Differences calculated by comparing the cumulative monthly coverage for each pandemic cohort to the cumulative coverage in the analogous month for the pre-pandemic cohort. Coverage for the pre-pandemic cohort ranged from 76.6% (76.2-76.9 at end of Grade 6 school year) to 81.4% (81.1-81.7 at 48 months of follow-up) for HPV, and 85.6% (end of Grade 6 year) to 88.9% (at 48 months of follow-up) for HB.

Table S1. Alberta's provincial immunization schedule. Each bullet represents one vaccine dose unless otherwise stated.

**Age**	**Vaccine**
2 months	• DTaP-IPV-HiB-HB[1]<br>• Pneumococcal conjugate<br>• Rotavirus
4 months	• DTaP-IPV-HiB-HB[1]<br>• Pneumococcal conjugate<br>• Meningococcal conjugate<br>• Rotavirus
6 months	• DTaP-IPV-HiB-HB[1]<br>• Pneumococcal conjugate (for high-risk children only)
6 months and older	• Influenza (annually)<br>• COVID-19
12 months	• MMRV[2]<br>• Meningococcal conjugate<br>• Pneumococcal conjugate
18 months	• DTaP-IPV-Hib<br>• MMRV[2]
4 years	• Tdap-IPV[3]
Grade 6	• HPV[4] (2 doses)<br>• HB (2 doses)
Grade 9	• Tdap[5]<br>• MenC-ACYW[6]
Adults	• Tdap[5] (every 10 years and one dose in pregnancy)<br>• Pneumococcal conjugate (one dose – 65 years and older)

[1]Diphtheria, tetanus, acellular pertussis, polio, haemophilus influenzae type b, hepatitis b

[2]Measles, mumps, rubella, and varicella

[3]Tetanus, diphtheria, acellular pertussis, polio

[4]Human papillomavirus

[5]Tetanus, diphtheria, acellular pertussis

[6]Meningococcal Conjugate Vaccine (Groups A, C, W-135 and Y)

Table S2. Monthly cumulative coverage for HPV vaccine for all cohorts, and difference in monthly coverage between pre-pandemic (Grade 5; 2017-18) and pandemic cohorts (Grade 6; 2019-20, 2020-21, 2021-22, 2022-23). Month zero represents end of grade year (July) relevant to each cohort.

Months from end of school year	Pre-pandemic cohort	Pandemic cohorts							
	2017-18	2019-20		2020-21		2021-22		2022-23	
	Coverage, % (95% CI)	Coverage, % (95% CI)	Difference, % (95% CI)	Coverage, % (95% CI)	Difference, % (95% CI)	Coverage, % (95% CI)	Difference, % (95% CI)	Coverage, % (95% CI)	Difference, % (95% CI)
0	65.61 (65.20, 66.02)	5.61 (5.42, 5.80)	-60.00 (-60.45, -59.55)	6.57 (6.36, 6.78)	-59.04 (-59.50, -58.59)	60.47 (60.06, 60.88)	-5.14 (-5.72, -4.56)	63.02 (62.62, 63.42)	-2.59 (-3.16, -2.02)
1	66.35 (65.95, 66.75)	6.74 (6.53, 6.95)	-59.61 (-60.07, -59.16)	8.96 (8.72, 9.19)	-57.39 (-57.86, -56.92)	61.21 (60.80, 61.62)	-5.14 (-5.72, -4.57)	63.43 (63.03, 63.83)	-2.92 (-3.48, -2.35)
2	66.85 (66.45, 67.26)	7.37 (7.15, 7.59)	-59.48 (-59.94, -59.03)	10.78 (10.52, 11.03)	-56.08 (-56.56, -55.60)	62.03 (61.62, 62.44)	-4.83 (-5.40, -4.25)	63.62 (63.22, 64.01)	-3.24 (-3.80, -2.67)
3	68.22 (67.82, 68.62)	15.63 (15.32, 15.93)	-52.60 (-53.10, -52.09)	15.09 (14.79, 15.39)	-53.13 (-53.63, -52.64)	63.04 (62.64, 63.45)	-5.18 (-5.75, -4.61)	64.55 (64.16, 64.95)	-3.67 (-4.23, -3.11)
4	69.28 (68.89, 69.67)	31.03 (30.64, 31.42)	-38.25 (-38.80, -37.70)	27.88 (27.50, 28.25)	-41.40 (-41.95, -40.86)	64.36 (63.95, 64.76)	-4.92 (-5.49, -4.36)	65.87 (65.48, 66.26)	-3.41 (-3.97, -2.85)
5	69.67 (69.28, 70.06)	32.50 (32.10, 32.89)	-37.17 (-37.73, -36.62)	38.03 (37.63, 38.44)	-31.64 (-32.20, -31.08)	65.23 (64.83, 65.63)	-4.44 (-5.00, -3.88)	66.78 (66.39, 67.17)	-2.89 (-3.45, -2.34)
6	70.23 (69.84, 70.62)	37.54 (37.13, 37.95)	-32.69 (-33.26, -32.13)	42.83 (42.42, 43.24)	-27.40 (-27.97, -26.84)	66.34 (65.95, 66.74)	-3.89 (-4.45, -3.33)	67.65 (67.26, 68.04)	-2.58 (-3.13, -2.03)
7	70.71 (70.32, 71.10)	42.24 (41.82, 42.65)	-28.47 (-29.04, -27.91)	47.30 (46.88, 47.71)	-23.41 (-23.98, -22.85)	67.63 (67.24, 68.02)	-3.08 (-3.64, -2.53)	68.60 (68.22, 68.99)	-2.11 (-2.66, -1.56)
8	71.05 (70.66, 71.43)	44.80 (44.39, 45.22)	-26.24 (-26.81, -25.68)	51.84 (51.42, 52.25)	-19.21 (-19.78, -18.64)	68.83 (68.44, 69.22)	-2.22 (-2.77, -1.67)	69.39 (69.01, 69.77)	-1.66 (-2.20, -1.12)

Months from end of school year	Pre-pandemic cohort	Pandemic cohorts							
	2017-18	2019-20		2020-21		2021-22		2022-23	
	Coverage, % (95% CI)	Coverage, % (95% CI)	Difference, % (95% CI)	Coverage, % (95% CI)	Difference, % (95% CI)	Coverage, % (95% CI)	Difference, % (95% CI)	Coverage, % (95% CI)	Difference, % (95% CI)
9	71.54 (71.15, 71.92)	46.20 (45.78, 46.62)	-25.34 (-25.91, -24.77)	53.73 (53.31, 54.14)	-17.81 (-18.38, -17.24)	69.45 (69.07, 69.84)	-2.09 (-2.63, -1.54)	69.94 (69.56, 70.32)	-1.60 (-2.14, -1.06)
10	72.12 (71.74, 72.50)	46.58 (46.16, 47.00)	-25.54 (-26.11, -24.98)	56.10 (55.69, 56.51)	-16.02 (-16.58, -15.46)	70.45 (70.06, 70.83)	-1.67 (-2.21, -1.13)	70.37 (69.99, 70.75)	-1.75 (-2.29, -1.21)
11	72.39 (72.01, 72.78)	47.07 (46.65, 47.49)	-25.32 (-25.89, -24.76)	59.52 (59.11, 59.93)	-12.88 (-13.43, -12.32)	70.99 (70.61, 71.37)	-1.40 (-1.94, -0.86)	70.69 (70.31, 71.06)	-1.71 (-2.24, -1.17)
12	72.49 (72.11, 72.88)	47.29 (46.87, 47.71)	-25.21 (-25.77, -24.64)	60.12 (59.71, 60.53)	-12.37 (-12.93, -11.81)	71.12 (70.74, 71.50)	-1.37 (-1.91, -0.83)	70.78 (70.40, 71.16)	-1.71 (-2.25, -1.18)
13	72.56 (72.18, 72.94)	49.02 (48.60, 49.44)	-23.54 (-24.11, -22.97)	60.66 (60.25, 61.07)	-11.90 (-12.45, -11.34)	71.25 (70.87, 71.63)	-1.31 (-1.85, -0.77)	-	-
14	72.61 (72.23, 72.99)	49.76 (49.34, 50.17)	-22.85 (-23.42, -22.28)	60.89 (60.48, 61.29)	-11.72 (-12.28, -11.16)	71.35 (70.97, 71.73)	-1.25 (-1.79, -0.71)	-	-
15	72.74 (72.36, 73.12)	50.90 (50.48, 51.31)	-21.84 (-22.41, -21.27)	61.43 (61.02, 61.83)	-11.31 (-11.86, -10.75)	71.56 (71.18, 71.94)	-1.18 (-1.72, -0.64)	-	-
16	72.90 (72.52, 73.27)	52.73 (52.31, 53.15)	-20.16 (-20.73, -19.60)	62.13 (61.72, 62.53)	-10.77 (-11.32, -10.22)	71.94 (71.56, 72.32)	-0.96 (-1.49, -0.42)	-	-
17	73.09 (72.71, 73.47)	55.95 (55.53, 56.36)	-17.14 (-17.70, -16.58)	62.58 (62.18, 62.99)	-10.50 (-11.06, -9.95)	72.20 (71.82, 72.57)	-0.89 (-1.42, -0.35)	-	-
18	73.24 (72.86, 73.62)	58.66 (58.25, 59.07)	-14.58 (-15.14, -14.02)	63.05 (62.64, 63.45)	-10.19 (-10.75, -9.64)	72.45 (72.07, 72.83)	-0.79 (-1.32, -0.26)	-	-

Months from end of school year	Pre-pandemic cohort	Pandemic cohorts							
	2017-18	2019-20		2020-21		2021-22		2022-23	
	Coverage, % (95% CI)	Coverage, % (95% CI)	Difference, % (95% CI)	Coverage, % (95% CI)	Difference, % (95% CI)	Coverage, % (95% CI)	Difference, % (95% CI)	Coverage, % (95% CI)	Difference, % (95% CI)
19	73.50 (73.13, 73.88)	63.01 (62.60, 63.41)	-10.50 (-11.05, -9.94)	63.84 (63.44, 64.24)	-9.66 (-10.21, -9.11)	72.68 (72.31, 73.06)	-0.82 (-1.35, -0.29)	-	-
20	73.65 (73.27, 74.03)	66.17 (65.77, 66.57)	-7.48 (-8.03, -6.94)	64.53 (64.13, 64.93)	-9.12 (-9.67, -8.57)	72.90 (72.52, 73.27)	-0.75 (-1.28, -0.22)	-	-
21	73.66 (73.29, 74.04)	66.99 (66.59, 67.38)	-6.68 (-7.22, -6.13)	64.93 (64.53, 65.33)	-8.73 (-9.28, -8.19)	73.06 (72.69, 73.43)	-0.60 (-1.13, -0.07)	-	-
22	73.67 (73.30, 74.05)	67.64 (67.25, 68.03)	-6.03 (-6.58, -5.49)	65.57 (65.18, 65.97)	-8.10 (-8.64, -7.55)	73.20 (72.83, 73.57)	-0.47 (-1.00, 0.06)	-	-
23	73.69 (73.31, 74.06)	68.43 (68.04, 68.82)	-5.26 (-5.80, -4.72)	65.86 (65.47, 66.26)	-7.83 (-8.37, -7.28)	73.31 (72.94, 73.68)	-0.38 (-0.91, 0.15)	-	-
24	73.71 (73.34, 74.09)	68.75 (68.36, 69.14)	-4.96 (-5.50, -4.42)	65.96 (65.57, 66.36)	-7.75 (-8.29, -7.20)	73.36 (72.99, 73.73)	-0.35 (-0.88, 0.18)	-	-
25	73.73 (73.35, 74.10)	69.07 (68.69, 69.46)	-4.65 (-5.19, -4.11)	66.06 (65.66, 66.45)	-7.67 (-8.21, -7.12)	-	-	-	-
26	73.74 (73.37, 74.12)	69.21 (68.82, 69.60)	-4.53 (-5.07, -3.99)	66.15 (65.75, 66.54)	-7.60 (-8.14, -7.05)	-	-	-	-
27	73.78 (73.40, 74.15)	69.60 (69.21, 69.98)	-4.18 (-4.72, -3.64)	66.52 (66.13, 66.91)	-7.26 (-7.80, -6.71)	-	-	-	-
28	73.84 (73.46, 74.21)	70.70 (70.32, 71.08)	-3.14 (-3.68, -2.61)	67.58 (67.19, 67.97)	-6.26 (-6.80, -5.72)	-	-	-	-

Months from end of school year	Pre-pandemic cohort	Pandemic cohorts							
	2017-18	2019-20		2020-21		2021-22		2022-23	
	Coverage, % (95% CI)	Coverage, % (95% CI)	Difference, % (95% CI)	Coverage, % (95% CI)	Difference, % (95% CI)	Coverage, % (95% CI)	Difference, % (95% CI)	Coverage, % (95% CI)	Difference, % (95% CI)
29	73.87 (73.49, 74.24)	71.44 (71.06, 71.82)	-2.43 (-2.96, -1.89)	68.33 (67.95, 68.72)	-5.53 (-6.07, -5.00)	-	-	-	-
30	73.89 (73.51, 74.26)	72.38 (72.01, 72.76)	-1.50 (-2.03, -0.97)	69.23 (68.85, 69.61)	-4.66 (-5.19, -4.12)	-	-	-	-
31	73.92 (73.54, 74.29)	73.44 (73.07, 73.81)	-0.48 (-1.01, 0.05)	70.23 (69.85, 70.61)	-3.68 (-4.22, -3.15)	-	-	-	-
32	73.94 (73.57, 74.32)	74.09 (73.72, 74.46)	0.14 (-0.38, 0.67)	70.86 (70.49, 71.24)	-3.08 (-3.61, -2.55)	-	-	-	-
33	73.98 (73.60, 74.35)	74.40 (74.04, 74.77)	0.42 (-0.10, 0.95)	71.50 (71.12, 71.87)	-2.48 (-3.01, -1.95)	-	-	-	-
34	74.00 (73.62, 74.37)	75.01 (74.64, 75.37)	1.01 (0.49, 1.53)	72.13 (71.76, 72.50)	-1.86 (-2.39, -1.34)	-	-	-	-
35	74.01 (73.64, 74.39)	75.51 (75.15, 75.87)	1.50 (0.98, 2.02)	72.72 (72.35, 73.09)	-1.29 (-1.82, -0.77)	-	-	-	-
36	74.02 (73.64, 74.39)	75.64 (75.28, 76.00)	1.62 (1.10, 2.14)	72.85 (72.48, 73.22)	-1.17 (-1.70, -0.64)	-	-	-	-
37	74.05 (73.68, 74.43)	75.84 (75.48, 76.20)	1.78 (1.26, 2.30)	-	-	-	-	-	-
38	74.08 (73.70, 74.45)	75.93 (75.57, 76.28)	1.85 (1.33, 2.37)	-	-	-	-	-	-

Months from end of school year	Pre-pandemic cohort	Pandemic cohorts							
	2017-18	2019-20		2020-21		2021-22		2022-23	
	Coverage, % (95% CI)	Coverage, % (95% CI)	Difference, % (95% CI)	Coverage, % (95% CI)	Difference, % (95% CI)	Coverage, % (95% CI)	Difference, % (95% CI)	Coverage, % (95% CI)	Difference, % (95% CI)
39	74.11 (73.73, 74.48)	75.99 (75.64, 76.35)	1.89 (1.37, 2.41)	-	-	-	-	-	-
40	74.22 (73.85, 74.60)	76.09 (75.73, 76.45)	1.87 (1.35, 2.39)	-	-	-	-	-	-
41	74.35 (73.98, 74.72)	76.13 (75.78, 76.49)	1.79 (1.27, 2.30)	-	-	-	-	-	-
42	74.45 (74.08, 74.82)	76.20 (75.84, 76.56)	1.75 (1.23, 2.27)	-	-	-	-	-	-
43	74.74 (74.37, 75.11)	76.28 (75.92, 76.64)	1.54 (1.03, 2.06)	-	-	-	-	-	-
44	74.92 (74.55, 75.29)	76.35 (75.99, 76.70)	1.42 (0.91, 1.94)	-	-	-	-	-	-
45	75.03 (74.66, 75.40)	76.40 (76.04, 76.76)	1.37 (0.86, 1.88)	-	-	-	-	-	-
46	75.20 (74.83, 75.57)	76.43 (76.07, 76.78)	1.22 (0.71, 1.74)	-	-	-	-	-	-
47	75.36 (74.99, 75.72)	76.46 (76.11, 76.82)	1.11 (0.59, 1.62)	-	-	-	-	-	-
48	75.40 (75.03, 75.76)	76.49 (76.14, 76.85)	1.10 (0.58, 1.61)	-	-	-	-	-	-

Months from end of school year	Pre-pandemic cohort	Pandemic cohorts							
	2017-18	2019-20		2020-21		2021-22		2022-23	
	Coverage, % (95% CI)	Coverage, % (95% CI)	Difference, % (95% CI)	Coverage, % (95% CI)	Difference, % (95% CI)	Coverage, % (95% CI)	Difference, % (95% CI)	Coverage, % (95% CI)	Difference, % (95% CI)
49	75.47 (75.10, 75.84)	-	-	-	-	-	-	-	-
50	75.52 (75.15, 75.89)	-	-	-	-	-	-	-	-
51	75.54 (75.18, 75.91)	-	-	-	-	-	-	-	-
52	75.56 (75.19, 75.93)	-	-	-	-	-	-	-	-
53	75.58 (75.21, 75.94)	-	-	-	-	-	-	-	-
54	75.60 (75.23, 75.96)	-	-	-	-	-	-	-	-
55	75.62 (75.26, 75.99)	-	-	-	-	-	-	-	-
56	75.65 (75.28, 76.01)	-	-	-	-	-	-	-	-
57	75.66 (75.29, 76.03)	-	-	-	-	-	-	-	-
58	75.68 (75.31, 76.04)	-	-	-	-	-	-	-	-

Months from end of school year	Pre-pandemic cohort	Pandemic cohorts							
	2017-18	2019-20		2020-21		2021-22		2022-23	
	Coverage, % (95% CI)	Coverage, % (95% CI)	Difference, % (95% CI)	Coverage, % (95% CI)	Difference, % (95% CI)	Coverage, % (95% CI)	Difference, % (95% CI)	Coverage, % (95% CI)	Difference, % (95% CI)
59	75.70 (75.33, 76.07)	-	-	-	-	-	-	-	-
60	75.71 (75.35, 76.08)	-	-	-	-	-	-	-	-
61	75.74 (75.37, 76.10)	-	-	-	-	-	-	-	-
62	75.75 (75.38, 76.12)	-	-	-	-	-	-	-	-
63	75.77 (75.40, 76.13)	-	-	-	-	-	-	-	-
64	75.79 (75.42, 76.15)	-	-	-	-	-	-	-	-
65	75.80 (75.43, 76.16)	-	-	-	-	-	-	-	-
66	75.90 (75.53, 76.26)	-	-	-	-	-	-	-	-
67	75.91 (75.55, 76.28)	-	-	-	-	-	-	-	-
68	75.93 (75.56, 76.29)	-	-	-	-	-	-	-	-

Months from end of school year	Pre-pandemic cohort	Pandemic cohorts							
	2017-18	2019-20		2020-21		2021-22		2022-23	
	Coverage, % (95% CI)	Coverage, % (95% CI)	Difference, % (95% CI)	Coverage, % (95% CI)	Difference, % (95% CI)	Coverage, % (95% CI)	Difference, % (95% CI)	Coverage, % (95% CI)	Difference, % (95% CI)
69	75.95 (75.58, 76.31)	-	-	-	-	-	-	-	-
70	75.96 (75.60, 76.33)	-	-	-	-	-	-	-	-
71	75.98 (75.61, 76.35)	-	-	-	-	-	-	-	-
72	75.99 (75.63, 76.36)	-	-	-	-	-	-	-	-

Table S3. Monthly cumulative coverage for HB vaccine for all cohorts, and difference in monthly coverage between pre-pandemic (Grade 5; 2017-18) and pandemic (Grade 6; 2019-20, 2020-21, 2021-22, 2022-23). Month zero represents end of grade year (July) relevant to each cohort.

Months from end of school year	Pandemic cohort	Pre-pandemic cohorts							
	2017-18	2019-20		2020-21		2021-22		2022-23	
	Coverage, % (95% CI)	Coverage, % (95% CI)	Difference, % (95% CI)	Coverage, % (95% CI)	Difference, % (95% CI)	Coverage, % (95% CI)	Difference, % (95% CI)	Coverage, % (95% CI)	Difference, % (95% CI)
0	73.72 (73.34, 74.09)	31.98 (31.59, 32.37)	-41.74 (-42.28, -41.20)	32.66 (32.28, 33.05)	-41.05 (-41.59, -40.51)	69.72 (69.33, 70.10)	-4.00 (-4.54, -3.46)	72.56 (72.19, 72.93)	-1.16 (-1.69, -0.63)
1	74.36 (73.98, 74.73)	32.97 (32.58, 33.37)	-41.38 (-41.93, -40.84)	34.61 (34.22, 35.01)	-39.74 (-40.29, -39.20)	70.30 (69.92, 70.69)	-4.06 (-4.59, -3.52)	72.92 (72.55, 73.28)	-1.44 (-1.97, -0.92)
2	74.80 (74.43, 75.17)	33.57 (33.17, 33.96)	-41.23 (-41.77, -40.69)	36.09 (35.69, 36.49)	-38.71 (-39.25, 38.16)	70.94 (70.56, 71.33)	-3.85 (-4.38, -3.32)	73.09 (72.72, 73.45)	-1.71 (-2.23, -1.19)
3	76.00 (75.63, 76.36)	39.67 (39.26, 40.08)	-36.32 (-36.87, -35.77)	39.33 (38.92, 39.73)	-39.67 (-37.22, -36.12)	71.72 (71.35, 72.10)	-4.27 (-4.80, -3.75)	73.77 (73.40, 74.13)	-2.23 (-2.74, -1.71)
4	76.97 (76.61, 77.33)	50.40 (49.98, 50.82)	-26.57 (-27.12, -26.02)	48.31 (47.90, 48.73)	-28.66 (-29.21, -28.11)	72.82 (72.45, 73.20)	-4.15 (-4.66, -3.63)	74.63 (74.27, 74.99)	-2.34 (-2.85, -1.83)
5	77.29 (76.94, 77.65)	51.53 (51.11, 51.95)	-25.76 (-26.32, -25.21)	55.31 (54.90, 55.73)	-21.98 (-22.53, -21.43)	73.48 (73.10, 73.85)	-3.82 (-4.34, -3.30)	75.26 (74.90, 75.62)	-2.03 (-2.54, -1.53)
6	77.81 (77.45, 78.16)	55.05 (54.64, 55.47)	-22.75 (-23.30, -22.21)	58.41 (58.00, 58.82)	-19.40 (-19.94, -18.86)	74.28 (73.91, 74.65)	-3.53 (-4.04, -3.02)	76.07 (75.72, 76.43)	-1.74 (-2.24, -1.24)
7	78.28 (77.93, 78.64)	58.27 (57.86, 58.69)	-20.01 (-20.55, -19.47)	61.48 (61.08, 61.88)	-16.80 (-17.34, -16.27)	75.18 (74.81, 75.54)	-3.11 (-3.61, -2.60)	76.74 (76.39, 77.09)	-1.54 (-2.04, -1.04)
8	78.61 (78.26, 78.97)	60.13 (59.72, 60.54)	-18.49 (-19.03, -17.95)	64.86 (64.46, 65.25)	-13.76 (-14.29, -13.23)	76.01 (75.65, 76.37)	-2.61 (-3.11, -2.11)	77.29 (76.95, 77.64)	-1.32 (-1.91, -0.83)

Months from end of school year	Pandemic cohort	Pre-pandemic cohorts							
	2017-18	2019-20		2020-21		2021-22		2022-23	
	Coverage, % (95% CI)	Coverage, % (95% CI)	Difference, % (95% CI)	Coverage, % (95% CI)	Difference, % (95% CI)	Coverage, % (95% CI)	Difference, % (95% CI)	Coverage, % (95% CI)	Difference, % (95% CI)
9	79.11 (78.76, 79.45)	61.14 (60.73, 61.55)	-17.97 (-18.50, -17.43)	66.25 (65.86, 66.65)	-12.85 (-13.38, -12.33)	76.45 (76.09, 76.81)	-2.66 (-3.15, -2.16)	77.67 (77.32, 78.01)	-1.44 (-1.93, -0.95)
10	79.66 (79.31, 80.00)	61.43 (61.02, 61.83)	-18.23 (-18.77, -17.70)	67.88 (67.49, 68.27)	-11.78 (-12.30, -11.26)	77.22 (76.87, 77.58)	-2.44 (-2.93, -1.94)	77.98 (77.63, 78.32)	-1.68 (-2.16, -1.20)
11	79.88 (79.54, 80.23)	61.81 (61.40, 62.22)	-18.07 (-18.61, -17.54)	70.42 (70.04, 70.80)	-9.47 (-9.98, -8.96)	77.65 (77.30, 78.00)	-2.24 (-2.73, -1.75)	78.22 (77.88, 78.56)	-1.67 (-2.15, -1.18)
12	79.97 (79.63, 80.31)	61.98 (61.58, 62.39)	-17.99 (-18.52, -17.46)	70.81 (70.43, 71.19)	-9.16 (-9.67, -8.65)	77.78 (77.43, 78.13)	-2.19 (-2.68, -1.70)	78.29 (77.95, 78.63)	-1.68 (-2.17, -1.20)
13	80.05 (79.71, 80.39)	63.38 (62.97, 63.78)	-16.67 (-17.20, -16.14)	71.16 (70.79, 71.54)	-8.89 (-9.39, -8.38)	77.89 (77.54, 78.24)	-2.16 (-2.65, -1.68)	-	-
14	80.14 (79.80, 80.48)	64.00 (63.60, 64.41)	-16.13 (-16.66, -15.61)	71.32 (70.95, 71.70)	-8.81 (-9.32, -8.31)	77.97 (77.62, 78.32)	-2.17 (-2.66, -1.68)	-	-
15	80.29 (79.95, 80.63)	64.83 (64.43, 65.23)	-15.46 (-15.99, -14.94)	71.78 (71.40, 72.15)	-8.52 (-9.02, -8.01)	78.11 (77.76, 78.46)	-2.18 (-2.66, -1.69)	-	-
16	80.50 (80.16, 80.84)	66.12 (65.73, 66.52)	-14.37 (-14.90, -13.85)	72.31 (71.94, 72.68)	-8.19 (-8.69, -7.69)	78.38 (78.03, 78.73)	-2.12 (-2.60, -1.63)	-	-
17	80.69 (80.35, 81.02)	68.47 (68.08, 68.86)	-12.22 (-12.73, -11.70)	72.67 (72.30, 73.04)	-8.01 (-8.51, -7.51)	78.55 (78.21, 78.90)	-2.13 (-2.62, -1.65)	-	-
18	80.86 (80.53, 81.20)	70.13 (69.75, 70.52)	-10.73 (-11.24, -10.22)	73.01 (72.65, 73.38)	-7.85 (-8.35, -7.35)	79.07 (78.73, 79.41)	-1.80 (-2.27, -1.32)	-	-

Months from end of school year	Pandemic cohort	Pre-pandemic cohorts							
	2017-18	2019-20		2020-21		2021-22		2022-23	
	Coverage, % (95% CI)	Coverage, % (95% CI)	Difference, % (95% CI)	Coverage, % (95% CI)	Difference, % (95% CI)	Coverage, % (95% CI)	Difference, % (95% CI)	Coverage, % (95% CI)	Difference, % (95% CI)
19	81.12 (80.79, 81.46)	73.00 (72.63, 73.38)	-8.12 (-8.62, -7.62)	73.61 (73.25, 73.98)	-7.51 (-8.00, -7.01)	79.28 (78.94, 79.63)	-1.84 (-2.31, -1.36)	-	-
20	81.26 (80.93, 81.59)	75.11 (74.75, 75.47)	-6.15 (-6.64, -5.66)	74.10 (73.73, 74.46)	-7.16 (-7.65, -6.67)	79.44 (79.10, 79.78)	-1.82 (-2.29, -1.34)	-	-
21	81.27 (80.94, 81.60)	75.73 (75.37, 76.09)	-5.54 (-6.03, -5.05)	74.38 (74.02, 74.74)	-6.89 (-7.38, -6.40)	79.58 (79.24, 79.92)	-1.69 (-2.16, -1.21)	-	-
22	81.28 (80.95, 81.62)	76.25 (75.89, 76.61)	-5.03 (-5.52, -4.54)	74.88 (74.52, 75.24)	-6.40 (-6.89, -5.91)	79.68 (79.34, 80.02)	-1.60 (-2.08, -1.13)	-	-
23	81.30 (80.97, 81.63)	76.84 (76.49, 77.20)	-4.46 (-4.94, -3.97)	75.11 (74.75, 75.47)	-6.19 (-6.68, -5.70)	79.77 (79.43, 80.11)	-1.53 (-2.01, -1.06)	-	-
24	81.33 (81.00, 81.66)	77.08 (76.72, 77.43)	-4.25 (-4.74, -3.77)	75.20 (74.84, 75.56)	-6.13 (-6.62, -5.64)	79.80 (79.46, 80.14)	-1.53 (-2.00, -1.05)	-	-
25	81.34 (81.00, 81.67)	77.28 (76.92, 77.63)	-4.06 (-4.55, -3.58)	75.27 (74.91, 75.63)	-6.07 (-6.56, -5.58)	-	-	-	-
26	81.34 (81.01, 81.68)	77.38 (77.03, 77.73)	-3.96 (-4.44, -3.48)	75.33 (74.97, 75.69)	-6.01 (-6.50, -5.52)	-	-	-	-
27	81.38 (81.04, 81.71)	77.64 (77.29, 77.99)	-3.73 (-4.21, -3.25)	75.65 (75.29, 76.00)	-5.73 (-6.22, -5.24)	-	-	-	-
28	81.45 (81.12, 81.78)	78.43 (78.08, 78.77)	-3.02 (-3.50, -3.54)	76.39 (76.04, 76.74)	-5.06 (-5.54, -4.57)	-	-	-	-

Months from end of school year	Pandemic cohort	Pre-pandemic cohorts							
	2017-18	2019-20		2020-21		2021-22		2022-23	
	Coverage, % (95% CI)	Coverage, % (95% CI)	Difference, % (95% CI)	Coverage, % (95% CI)	Difference, % (95% CI)	Coverage, % (95% CI)	Difference, % (95% CI)	Coverage, % (95% CI)	Difference, % (95% CI)
29	81.47 (81.14, 81.80)	78.93 (78.59, 79.27)	-2.54 (-3.02, -2.06)	76.88 (76.52, 77.23)	-4.59 (-5.08, -4.11)	-	-	-	-
30	81.49 (81.16, 81.82)	79.57 (79.24, 79.91)	-1.92 (-2.39, -1.44)	77.59 (77.24, 77.94)	-3.90 (-4.38, -3.42)	-	-	-	-
31	81.52 (81.19, 81.85)	80.34 (80.00, 80.67)	-1.18 (-1.65, -0.71)	78.29 (77.94, 78.63)	-3.23 (-3.71, -2.76)	-	-	-	-
32	81.54 (81.21, 81.87)	80.82 (80.49, 81.15)	-0.72 (-1.19, -0.25)	78.70 (78.35, 79.04)	-2.85 (-3.32, -2.37)	-	-	-	-
33	81.57 (81.24, 81.90)	81.01 (80.69, 81.34)	-0.56 (-1.02, -0.09)	79.06 (78.72, 79.39)	-2.51 (-2.99, -2.04)	-	-	-	-
34	81.58 (81.25, 81.91)	81.42 (81.09, 81.75)	-0.16 (-0.63, 0.30)	79.45 (79.11, 79.78)	-2.13 (-2.61, -1.66)	-	-	-	-
35	81.60 (81.27, 81.93)	81.70 (81.38, 82.02)	0.10 (-0.36, 0.56)	79.82 (79.48, 80.15)	-1.78 (-2.25, -1.66)	-	-	-	-
36	81.61 (81.28, 81.94)	81.78 (81.46, 82.11)	0.17 (-0.29, 0.64)	79.91 (79.57, 80.24)	-1.70 (-2.17, -1.23)	-	-	-	-
37	81.64 (81.31, 81.97)	81.90 (81.58, 82.22)	0.25 (-0.21, 0.72)	-	-	-	-	-	-
38	81.65 (81.32, 81.99)	81.95 (81.63, 82.28)	0.30 (-0.16, 0.76)	-	-	-	-	-	-

Months from end of school year	Pandemic cohort	Pre-pandemic cohorts							
	2017-18	2019-20		2020-21		2021-22		2022-23	
	Coverage, % (95% CI)	Coverage, % (95% CI)	Difference, % (95% CI)	Coverage, % (95% CI)	Difference, % (95% CI)	Coverage, % (95% CI)	Difference, % (95% CI)	Coverage, % (95% CI)	Difference, % (95% CI)
39	81.71 (81.38, 82.04)	82.01 (81.69, 82.34)	0.30 (-0.16, 0.76)	-	-	-	-	-	-
40	81.86 (81.53, 82.19)	82.08 (81.75, 82.40)	0.22 (-0.24, 0.68)	-	-	-	-	-	-
41	81.97 (81.65, 82.30)	82.10 (81.78, 82.42)	0.12 (-0.34, 0.58)	-	-	-	-	-	-
42	82.08 (81.76, 82.42)	82.35 (82.03, 82.67)	0.26 (-0.20, 0.72)	-	-	-	-	-	-
43	82.40 (82.08, 82.73)	82.41 (82.09, 82.73)	0.01 (-0.45, 0.47)	-	-	-	-	-	-
44	82.63 (82.30, 82.95)	82.46 (82.14, 82.78)	-0.17 (-0.62, 0.29)	-	-	-	-	-	-
45	82.72 (82.40, 83.05)	82.50 (82.18, 82.82)	-0.22 (-0.68, 0.23)	-	-	-	-	-	-
46	82.83 (82.51, 83.16)	82.53 (82.21, 82.84)	-0.31 (-0.76, 0.14)	-	-	-	-	-	-
47	82.97 (82.65, 83.29)	82.55 (82.23, 82.87)	-0.42 (-0.87, 0.03)	-	-	-	-	-	-
48	83.01 (82.69, 83.33)	82.58 (82.26, 82.90)	-0.43 (-0.88, 0.02)	-	-	-	-	-	-

Months from end of school year	Pandemic cohort	Pre-pandemic cohorts							
	2017-18	2019-20		2020-21		2021-22		2022-23	
	Coverage, % (95% CI)	Coverage, % (95% CI)	Difference, % (95% CI)	Coverage, % (95% CI)	Difference, % (95% CI)	Coverage, % (95% CI)	Difference, % (95% CI)	Coverage, % (95% CI)	Difference, % (95% CI)
49	83.07 (82.75, 83.39)	-	-	-	-	-	-	-	-
50	83.09 (82.77, 83.41)	-	-	-	-	-	-	-	-
51	83.13 (82.81, 83.45)	-	-	-	-	-	-	-	-
52	83.19 (82.87, 83.51)	-	-	-	-	-	-	-	-
53	83.21 (82.89, 83.53)	-	-	-	-	-	-	-	-
54	83.24 (82.92, 83.56)	-	-	-	-	-	-	-	-
55	83.29 (82.97, 83.61)	-	-	-	-	-	-	-	-
56	83.34 (83.03, 83.66)	-	-	-	-	-	-	-	-
57	83.37 (83.05, 83.69)	-	-	-	-	-	-	-	-
58	83.41 (83.09, 83.72)	-	-	-	-	-	-	-	-

Months from end of school year	Pandemic cohort	Pre-pandemic cohorts							
	2017-18	2019-20		2020-21		2021-22		2022-23	
	Coverage, % (95% CI)	Coverage, % (95% CI)	Difference, % (95% CI)	Coverage, % (95% CI)	Difference, % (95% CI)	Coverage, % (95% CI)	Difference, % (95% CI)	Coverage, % (95% CI)	Difference, % (95% CI)
59	83.45 (83.14, 83.77)	-	-	-	-	-	-	-	-
60	83.49 (83.17, 83.81)	-	-	-	-	-	-	-	-
61	83.54 (83.22, 83.85)	-	-	-	-	-	-	-	-
62	83.56 (83.25, 83.88)	-	-	-	-	-	-	-	-
63	83.58 (83.27, 83.90)	-	-	-	-	-	-	-	-
64	83.62 (83.30, 83.93)	-	-	-	-	-	-	-	-
65	83.64 (83.32, 83.95)	-	-	-	-	-	-	-	-
66	84.22 (83.91, 84.54)	-	-	-	-	-	-	-	-
67	84.24 (83.93, 84.56)	-	-	-	-	-	-	-	-
68	84.25 (83.94, 84.56)	-	-	-	-	-	-	-	-

Months from end of school year	Pandemic cohort	Pre-pandemic cohorts							
	2017-18	2019-20		2020-21		2021-22		2022-23	
	Coverage, % (95% CI)	Coverage, % (95% CI)	Difference, % (95% CI)	Coverage, % (95% CI)	Difference, % (95% CI)	Coverage, % (95% CI)	Difference, % (95% CI)	Coverage, % (95% CI)	Difference, % (95% CI)
69	84.27 (83.94, 84.56)	-	-	-	-	-	-	-	-
70	84.29 (83.98, 84.60)	-	-	-	-	-	-	-	-
71	84.31 (84.00, 84.62)	-	-	-	-	-	-	-	-
72	84.33 (84.02, 84.64)	-	-	-	-	-	-	-	-

Table S4. Monthly cumulative coverage for MenC-ACYW vaccine for all Grade 9 cohorts, and difference in monthly coverage between pre-pandemic and pandemic cohorts. Month zero represents end of grade year (July) relevant to each cohort.

Months from end of school year	Pre-pandemic cohort	Pandemic cohorts							
	2018-19	2019-20		2020-21		2021-22		2022-23	
	Coverage, % (95% CI)	Coverage, % (95% CI)	Difference, % (95% CI)	Coverage, % (95% CI)	Difference, % (95% CI)	Coverage, % (95% CI)	Difference, % (95% CI)	Coverage, % (95% CI)	Difference, % (95% CI)
0	84.87 (84.55, 85.19)	79.12 (78.76, 79.48)	-5.75 (-6.23, -5.26)	53.11 (52.68, 53.55)	-31.76 (-32.30, -31.21)	76.89 (76.53, 77.25)	-7.98 (-8.46, -7.50)	76.98 (76.64, 77.33)	-7.89 (-8.36, -7.41)
1	85.19 (84.87, 85.51)	78.82 (79.47, 80.18)	-5.36 (-5.84, -4.89)	57.93 (57.50, 58.36)	-27.25 (-27.79, -26.72)	77.56 (77.21, 77.92)	-7.62 (-8.10, -7.14)	77.46 (77.11, 77.80)	-7.73 (-8.20, -7.26)
2	85.32 (85.00, 85.64)	80.25 (79.90, 80.60)	-5.07 (-5.55, -4.60)	59.36 (58.93, 59.79)	-25.96 (-25.50, -25.43)	77.86 (77.51, 78.22)	-7.46 (-7.93, -6.98)	77.64 (77.29, 77.98)	-7.69 (-8.15, -7.22)
3	85.47 (85.16, 85.42)	80.42 (80.07, 80.77)	-5.05 (-5.53, -4.58)	60.02 (59.60, 60.45)	-25.45 (-25.98, -24.92)	78.02 (77.67, 78.37)	-7.45 (-7.92, -6.98)	77.79 (77.45, 78.14)	-7.68 (-8.15, -7.21)
4	85.60 (85.29, 85.92)	80.61 (80.26, 80.96)	-4.99 (-5.46, -4.52)	61.53 (61.10, 61.95)	-24.08 (-24.61, -23.55)	78.14 (77.79, 78.49)	-7.47 (-7.94, -6.99)	77.96 (77.61, 78.30)	-7.65 (-8.11, -7.18)
5	85.70 (95.38, 86.01)	80.77 (80.42, 81.12)	-4.92 (-5.39, -4.45)	62.12 (61.69, 62.54)	-23.58 (-24.11, -23.05)	78.24 (77.89, 78.59)	-7.46 (-7.93, -6.99)	78.05 (77.71, 78.40)	-7.65 (-8.11, -7.18)
6	85.78 (85.46, 86.09)	80.83 (80.48, 81.18)	-4.95 (-5.41, -4.48)	62.63 (62.20, 63.05)	-23.15 (-23.68, -22.63)	78.36 (78.01, 78.71)	-7.42 (-7.89, -6.95)	78.17 (77.83, 78.51)	-7.61 (-8.07, -7.14)
7	85.86 (85.55, 86.18)	80.96 (80.61, 81.30)	-4.91 (-5.38, -4.44)	63.59 (63.17, 64.01)	-22.27 (-22.80, -21.75)	78.63 (78.28, 78.97)	-7.24 (-7.71, -6.77)	78.27 (77.93, 78.61)	-7.59 (-8.06, -7.13)
8	85.92 (85.61, 86.23)	81.01 (80.66, 81.36)	-4.91 (-5.38, -4.44)	64.45 (64.03, 64.86)	-21.47 (-22.00, -20.95)	78.87 (78.52, 79.22)	-7.05 (-7.52, -6.58)	78.36 (78.02, 78.70)	-7.56 (-8.02, -7.09)

Months from end of school year	Pre-pandemic cohort	Pandemic cohorts							
	2018-19	2019-20		2020-21		2021-22		2022-23	
	Coverage, % (95% CI)	Coverage, % (95% CI)	Difference, % (95% CI)	Coverage, % (95% CI)	Difference, % (95% CI)	Coverage, % (95% CI)	Difference, % (95% CI)	Coverage, % (95% CI)	Difference, % (95% CI)
9	85.94 (85.63, 86.25)	81.10 (80.76, 81.45)	-4.84 (-5.30, -4.37)	65.07 (64.65, 65.49)	-20.87 (-21.39, -20.35)	79.03 (78.69, 79.38)	-6.91 (-7.37, -6.44)	78.43 (78.09, 78.77)	-7.51 (-7.97, -7.05)
10	85.96 (85.64, 85.27)	81.17 (80.82, 81.52)	-4.79 (-5.26, -4.32)	65.64 (65.23, 66.05)	-20.32 (-20.84, -19.80)	79.20 (78.86, 79.55)	-6.75 (-7.22, -6.29)	78.48 (78.14, 78.82)	-7.47 (-7.94, -7.01)
11	85.97 (85.66, 86.28)	81.21 (80.86, 81.56)	-4.76 (-5.23, -4.29)	66.06 (65.65, 66.47)	-19.91 (-20.43, -19.39)	79.46 (79.11, 79.80)	-6.51 (-6.98, -6.05)	78.53 (78.19, 78.87)	-7.44 (-7.90, -6.98)
12	85.98 (85.67, 86.29)	81.23 (80.89, 81.58)	-4.75 (-5.21, -4.28)	66.35 (65.94, 66.76)	-19.63 (-20.15, -19.12)	79.68 (79.34, 80.02)	-6.30 (-6.77, -5.84)	78.58 (78.24, 78.92)	-7.40 (-7.86, -6.94)
13	86.01 (85.69, 86.32)	81.40 (81.05, 81.74)	-4.61 (-5.07, -4.14)	66.75 (66.34, 67.16)	-19.26 (-19.77, -18.74)	79.91 (79.57, 80.25)	-6.10 (-6.56, -5.63)	-	-
14	86.03 (85.72, 86.34)	81.48 (81.13, 81.82)	-4.55 (-5.02, -4.09)	66.92 (66.51, 67.33)	-19.11 (-19.63, -18.60)	80.02 (79.68, 80.36)	-6.01 (-6.47, -5.55)	-	-
15	86.06 (85.74, 86.37)	81.53 (81.18, 81.87)	-4.53 (-4.99, -4.06)	67.05 (66.64, 67.46)	-19.00 (-19.52, -18.49)	80.09 (79.75, 80.43)	-5.97 (-6.43, -5.51)	-	-
16	86.07 (85.76, 86.38)	81.60 (81.25, 81.94)	-4.47 (-4.94, -4.01)	67.17 (66.76, 67.58)	-18.90 (-19.42, -18.39)	80.19 (79.85, 80.53)	-5.88 (-6.34, -5.42)	-	-
17	86.10 (85.79, 86.41)	81.68 (81.34, 82.02)	-4.42 (-4.88, -3.96)	67.28 (66.87, 67.69)	-18.82 (-19.34, -18.31)	80.24 (79.90, 80.57)	-5.86 (-6.32, -5.40)	-	-
18	85.12 (85.80, 86.43)	81.73 (81.39, 82.07)	-4.38 (-4.85, -3.92)	67.43 (67.02, 67.84)	-18.69 (-19.20, -18.17)	80.31 (79.97, 80.65)	-5.80 (-6.26, -5.34)	-	-

Months from end of school year	Pre-pandemic cohort	Pandemic cohorts							
	2018-19	2019-20		2020-21		2021-22		2022-23	
	Coverage, % (95% CI)	Coverage, % (95% CI)	Difference, % (95% CI)	Coverage, % (95% CI)	Difference, % (95% CI)	Coverage, % (95% CI)	Difference, % (95% CI)	Coverage, % (95% CI)	Difference, % (95% CI)
19	86.13 (85.82, 86.44)	81.79 (81.45, 82.13)	-4.34 (-4.80, -3.88)	67.82 (67.42, 68.23)	-18.30 (-18.82, -17.79)	80.38 (80.04, 80.72)	-5.75 (-6.21, -5.29)	-	-
20	86.15 (85.84, 86.46)	81.88 (81.53, 82.22)	-4.27 (-4.74, -3.81)	68.35 (67.94, 68.75)	-17.80 (-18.31, -17.29)	80.45 (80.11, 80.78)	-5.70 (-6.16, -5.25)	-	-
21	86.17 (85.86, 86.48)	81.94 (81.60, 82.28)	-4.23 (-4.69, -3.77)	68.84 (68.44, 69.25)	-17.32 (-17.83, -16.81)	80.50 (80.16, 80.83)	-5.67 (-6.13, -5.21)	-	-
22	86.19 (85.88, 86.50)	82.00 (81.66, 82.34)	-4.19 (-4.65, -3.73)	69.33 (68.93, 69.74)	-16.85 (-17.36, -16.35)	80.54 (80.20, 80.87)	-5.65 (-6.11, -5.19)	-	-
23	86.20 (85.89, 86.51)	82.08 (81.74, 82.42)	-4.12 (-4.58, -3.65)	69.93 (69.53, 70.33)	-16.27 (-16.77, -15.76)	80.57 (80.24, 80.91)	-5.62 (-6.08, -5.16)	-	-
24	86.21 (85.90, 86.52)	82.12 (81.78, 82.46)	-4.08 (-4.55, -3.62)	70.45 (70.05, 70.85)	-15.76 (-16.26, -15.25)	80.63 (80.29, 80.96)	-5.58 (-6.04, -5.12)	-	-
25	86.24 (85.93, 86.55)	82.18 (81.84, 82.52)	-4.06 (-4.52, -3.60)	71.15 (70.75, 71.54)	-15.09 (-15.59, -14.59)	-	-	-	-
26	86.26 (85.95, 86.57)	82.22 (81.88, 82.56)	-4.04 (-4.50, -3.58)	71.48 (71.08, 71.87)	-14.78 (-15.28, -14.28)	-	-	-	-
27	86.27 (85.96, 86.58)	82.27 (81.93, 82.61)	-4.00 (-4.46, -3.54)	71.62 (71.22, 72.01)	-14.66 (-15.16, -14.16)	-	-	-	-
28	86.29 (85.98, 86.60)	82.31 (81.97, 82.65)	-3.98 (-4.44, -3.52)	71.75 (71.36, 72.15)	-14.54 (-15.04, -14.04)	-	-	-	-

Months from end of school year	Pre-pandemic cohort	Pandemic cohorts							
	2018-19	2019-20		2020-21		2021-22		2022-23	
	Coverage, % (95% CI)	Coverage, % (95% CI)	Difference, % (95% CI)	Coverage, % (95% CI)	Difference, % (95% CI)	Coverage, % (95% CI)	Difference, % (95% CI)	Coverage, % (95% CI)	Difference, % (95% CI)
29	86.32 (86.01, 86.63)	82.33 (82.00, 82.67)	-3.99 (-4.45, -3.53)	71.85 (71.46, 72.24)	-14.48 (-14.97, -13.98)	-	-	-	-
30	86.34 (86.03, 86.65)	82.40 (82.06, 82.74)	-3.94 (-4.40, -3.48)	71.98 (71.58, 72.37)	-14.36 (-14.86, -13.86)	-	-	-	-
31	86.36 (86.05, 86.67)	82.48 (82.14, 82.82)	-3.88 (-4.34, -3.42)	72.08 (71.69, 72.47)	-14.28 (-14.78, -13.78)	-	-	-	-
32	86.39 (86.09, 86.70)	82.63 (82.29, 82.96)	-3.77 (-4.22, -3.31)	72.21 (71.82, 72.60)	-14.19 (-14.68, -13.69)	-	-	-	-
33	86.42 (86.11, 86.73)	82.77 (82.44, 83.11)	-3.65 (-4.10, -3.19)	72.27 (71.88, 72.66)	-14.14 (-14.64, -13.65)	-	-	-	-
34	86.43 (86.13, 86.74)	82.95 (82.62, 83.29)	-3.48 (-3.93, -3.03)	72.32 (71.93, 72.71)	-14.12 (-14.61, -13.62)	-	-	--	-
35	86.46 (86.16, 86.77)	83.14 (82.80, 83.47)	-3.33 (-3.78, -2.88)	72.39 (72.00, 72.78)	-14.07 (-14.57, -13.58)	-	-	-	-
36	86.47 (86.16, 86.78)	83.24 (82.91, 83.57)	-3.23 (-3.69, -2.78)	72.46 (72.07, 72.85)	-14.01 (-14.51, -13.51)	-	-	-	-
37	86.50 (86.19, 86.80)	83.31 (82.98, 83.64)	-3.18 (-3.63, -2.73)	-	-	-	-	-	-
38	86.50 (86.19, 86.81)	83.34 (83.01, 83.67)	-3.17 (-3.62, -2.72)	-	-	-	-	-	-

Months from end of school year	Pre-pandemic cohort	Pandemic cohorts							
	2018-19	2019-20		2020-21		2021-22		2022-23	
	Coverage, % (95% CI)	Coverage, % (95% CI)	Difference, % (95% CI)	Coverage, % (95% CI)	Difference, % (95% CI)	Coverage, % (95% CI)	Difference, % (95% CI)	Coverage, % (95% CI)	Difference, % (95% CI)
39	86.51 (86.20, 86.82)	83.35 (83.02, 83.68)	-3.17 (-3.62, -2.71)	-	-	-	-	-	-
40	86.52 (86.21, 86.83)	83.37 (83.04, 83.70)	-3.15 (-3.60, -2.70)	-	-	-	-	-	-
41	86.53 (86.23, 86.84)	83.39 (83.06, 83.72)	-3.15 (-3.60, -2.70)	-	-	-	-	-	-
42	86.54 (86.23, 86.85)	83.41 (83.08, 83.74)	-3.13 (-3.58, -2.68)	-	-	-	-	-	-
43	86.55 (86.24, 86.85)	83.42 (83.09, 83.75)	-3.13 (-3.58, -2.68)	-	-	-	-	-	-
44	86.56 (86.25, 86.87)	83.44 (83.11, 83.77)	-3.13 (-3.58, -2,68)	-	-	-	-	-	-
45	86.57 (86.26, 86.88)	83.44 (83.12, 83.77)	-3.14 (-3.59, -2.69)	-	-	-	-	-	-
46	86.58 (86.28, 86.89)	83.45 (83.12, 83.78)	-3.14 (-3.59. -2.69)	-	-	-	-	-	-
47	86.59 (86.28, 86.89)	83.45 (83.12, 83.78)	-3.14 (-3.59, -2.69)	-	-	-	-	-	-
48	86.60 (86.29, 86.90)	-	-	-	-	-	-	-	-

Months from end of school year	Pre-pandemic cohort	Pandemic cohorts							
	2018-19	2019-20		2020-21		2021-22		2022-23	
	Coverage, % (95% CI)	Coverage, % (95% CI)	Difference, % (95% CI)	Coverage, % (95% CI)	Difference, % (95% CI)	Coverage, % (95% CI)	Difference, % (95% CI)	Coverage, % (95% CI)	Difference, % (95% CI)
49	86.60 (86.30, 86.91)	-	-	-	-	-	-	-	-
50	86.61 (86.30, 86.91)	-	-	-	-	-	-	-	-
51	86.61 (86.30, 86.91)	-	-	-	-	-	-	-	-
52	86.61 (86.30, 86.92)	-	-	-	-	-	-	-	-
53	86.61 (86.30, 86.92)	-	-	-	-	-	-	-	-
54	86.63 (86.32, 86.94)	-	-	-	-	-	-	-	-
55	86.63 (86.32, 86.94)	-	-	-	-	-	-	-	-
56	86.63 (86.33, 86.94)	-	-	-	-	-	-	-	-
57	86.63 (86.33, 86.94)	-	-	-	-	-	-	-	-
58	86.63 (86.33, 86.94)	-	-	-	-	-	-	-	-

Months from end of school year	Pre-pandemic cohort	Pandemic cohorts							
	2018-19	2019-20		2020-21		2021-22		2022-23	
	Coverage, % (95% CI)	Coverage, % (95% CI)	Difference, % (95% CI)	Coverage, % (95% CI)	Difference, % (95% CI)	Coverage, % (95% CI)	Difference, % (95% CI)	Coverage, % (95% CI)	Difference, % (95% CI)
59	86.63 (86.33, 86.94)	-	-	-	-	-	-	-	-
60	86.63 (86.33, 86.94)	-	-	-	-	-	-	-	-

Table S5. Monthly cumulative coverage for Tdap vaccine for all Grade 9 cohorts, and difference in monthly coverage between pre-pandemic and pandemic cohorts. Month zero represents end of grade year (July) relevant to each cohort.

Months from end of school year	Pre-pandemic cohort	Pandemic cohorts							
	2018-19	2019-20		2020-21		2021-22		2022-23	
	Coverage, % (95% CI)	Coverage, % (95% CI)	Difference, % (95% CI)	Coverage, % (95% CI)	Difference, % (95% CI)	Coverage, % (95% CI)	Difference, % (95% CI)	Coverage, % (95% CI)	Difference, % (95% CI)
0	85.58 (85.26, 85.89)	80.22 (79.87, 80.58)	-5.35 (-5.83, -4.88)	55.12 (54.68, 55.55)	-30.46 (-31.00, -29.92)	77.52 (77.16, 77.87)	-8.06 (-8.53, -7.58)	77.62 (77.28, 77.97)	-7.95 (-8.42, -7.48)
1	85.90 (85.59, 86.21)	80.89 (80.54, 81.24)	-5.01 (-5.48, -4.54)	59.82 (59.39, 60.25)	-26.08 (-26.61, -25.55)	78.21 (77.86, 78.56)	-7.70 (-8.17, -7.23)	78.11 (77.76, 78.45)	-7.79 (-8.26, -7.33)
2	86.03 (85.72, 86.34)	81.31 (80.96, 81.65)	-4.72 (-5.19, -4.26)	61.25 (60.83, 61.68)	-24.78 (-25.31, -24.25)	78.51 (78.17, 78.86)	-7.52 (-7.98, -7.05)	78.3 (77.96, 78.64)	-7.73 (-8.20, -7.27)
3	86.18 (85.87, 86.49)	81.48 (81.14, 81.82)	-4.70 (-5.17, -4.24)	61.94 (61.52, 62.36)	-24.24 (-24.77, -23.72)	78.69 (78.35, 79.04)	-7.49 (-7.95, -7.02)	78.48 (78.14, 78.82)	-7.70 (-8.16, -7.24)
4	86.30 (85.99, 86.61)	81.69 (81.35, 82.04)	-4.61 (-5.07, -4.15)	63.4 (62.98, 63.82)	-22.90 (-23.42, -22.38)	78.83 (78.48, 79.17)	-7.47 (-7.94, -7.01)	78.65 (78.31, 78.99)	-7.65 (-8.11, -7.21)
5	86.41 (86.10, 86.72)	81.85 (81.51, 82.2)	-4.56 (-5.02, -4.10)	63.99 (63.57, 64.41)	-22.42 (-22.94, -21.90)	78.95 (78.6, 79.29)	-7.46 (-7.93, -7.00)	78.74 (78.4, 79.08)	-7.67 (-8.13, -7.21)
6	86.50 (86.19, 86.81)	81.92 (81.58, 82.26)	-4.58 (-5.04, -4.12)	64.5 (64.09, 64.92)	-22.00 (-22.52, -21.48)	79.06 (78.71, 79.4)	-7.45 (-7.91, -6.98)	78.83 (78.49, 79.17)	-7.67 (-8.13, -7.19)
7	86.59 (86.28, 86.90)	82.02 (81.68, 82.36)	-4.57 (-5.03, -4.11)	65.43 (65.02, 65.85)	-21.16 (-21.67, -20.64)	79.32 (78.98, 79.67)	-7.27 (-7.73, -6.81)	78.94 (78.61, 79.28)	-7.65 (-8.10, -7.14)
8	86.66 (86.35, 86.96)	82.09 (81.75, 82.43)	-4.57 (-5.03, -4.11)	66.3 (65.88, 66.71)	-20.36 (-20.87, -19.85)	79.57 (79.23, 79.91)	-7.09 (-7.54, -6.63)	79.06 (78.73, 79.4)	-7.59 (-8.05, -7.08)
9	86.69 (86.38, 86.99)	82.2 (81.86, 82.53)	-4.49 (-4.95, -4.11)	66.89 (66.48, 67.3)	-19.79 (-20.30, -19.28)	79.74 (79.4, 80.08)	-6.94 (-7.40, -6.48)	79.15 (78.82, 79.49)	-7.53 (-7.99, -7.04)

Months from end of school year	Pre-pandemic cohort	Pandemic cohorts							
	2018-19	2019-20		2020-21		2021-22		2022-23	
	Coverage, % (95% CI)	Coverage, % (95% CI)	Difference, % (95% CI)	Coverage, % (95% CI)	Difference, % (95% CI)	Coverage, % (95% CI)	Difference, % (95% CI)	Coverage, % (95% CI)	Difference, % (95% CI)
10	86.72 (86.41, 87.02)	82.28 (81.94, 82.62)	-4.44 (-4.89, -4.03)	67.47 (67.06, 67.87)	-19.25 (-19.76, -18.74)	79.93 (79.59, 80.27)	-6.79 (-7.24, -6.33)	79.22 (78.89, 79.56)	-7.49 (-7.95, -7.00)
11	86.77 (86.46, 87.07)	82.35 (82.01, 82.69)	-4.41 (-4.87, -3.98)	67.9 (67.49, 68.31)	-18.86 (-19.37, -18.36)	80.2 (79.86, 80.54)	-6.57 (-7.02, -6.11)	79.31 (78.97, 79.64)	-7.46 (-7.91, -6.96)
12	86.79 (86.49, 87.10)	82.41 (82.07, 82.75)	-4.39 (-4.84, -3.96)	68.23 (67.82, 68.64)	-18.57 (-19.07, -18.06)	80.45 (80.11, 80.79)	-6.35 (-6.80, -5.89)	79.39 (79.05, 79.72)	-7.41 (-7.86, -6.96)
13	86.84 (86.53, 87.14)	82.59 (82.25, 82.93)	-4.25 (-4.70, -3.93)	68.64 (68.24, 69.05)	-18.19 (-18.70, -17.69)	80.72 (80.39, 81.06)	-6.11 (-6.57, -5.66)	-	-
14	86.88 (86.57, 87.18)	82.67 (82.34, 83.01)	-4.21 (-4.66, -3.80)	68.85 (68.44, 69.25)	-18.03 (-18.53, -17.52)	80.86 (80.52, 81.19)	-6.02 (-6.47, -5.57)	-	-
15	86.93 (86.62, 87.23)	82.73 (82.39, 83.06)	-4.20 (-4.65, -3.75)	68.99 (68.59, 69.4)	-17.93 (-18.44, -17.43)	80.96 (80.62, 81.29)	-5.97 (-6.42, -5.52)	-	-
16	86.94 (86.64, 87.25)	82.81 (82.47, 83.14)	-4.14 (-4.59, -3.75)	69.13 (68.73, 69.54)	-17.81 (-18.32, -17.31)	81.09 (80.76, 81.42)	-5.86 (-6.31, -5.41)	-	-
17	86.97 (86.67, 87.28)	82.88 (82.55, 83.21)	-4.09 (-4.54, -3.69)	69.26 (68.85, 69.66)	-17.57 (-18.22, -17.21)	81.14 (80.81, 81.47)	-5.84 (-6.28, -5.39)	-	-
18	86.99 (86.69, 87.29)	82.94 (82.61, 83.27)	-4.05 (-4.50, -3.64)	69.42 (69.02, 69.82)	-17.17 (-18.07, -17.07)	81.22 (80.89, 81.55)	-5.77 (-6.22, -5.32)	-	-

Months from end of school year	Pre-pandemic cohort	Pandemic cohorts							
	2018-19	2019-20		2020-21		2021-22		2022-23	
	Coverage, % (95% CI)	Coverage, % (95% CI)	Difference, % (95% CI)	Coverage, % (95% CI)	Difference, % (95% CI)	Coverage, % (95% CI)	Difference, % (95% CI)	Coverage, % (95% CI)	Difference, % (95% CI)
19	87.01 (86.71, 87.32)	83.01 (82.68, 83.34)	-4.00 (-4.45, -3.60)	69.84 (69.44, 70.24)	-16.68 (-17.67, -16.67)	81.33 (81.0, 81.66)	-5.69 (-6.14, -5.24)	-	-
20	87.04 (86.74, 87.34)	83.1 (82.77, 83.44)	-3.93 (-4.38, -3.55)	70.35 (69.96, 70.75)	-16.20 (-17.18, -16.18)	81.41 (81.07, 81.74)	-5.63 (-6.08, -5.18)	-	-
21	87.07 (86.76, 87.37)	83.2 (82.87, 83.53)	-3.87 (-4.31, -3.49)	70.86 (70.47, 71.26)	-15.74 (-16.70, -15.71)	81.48 (81.15, 81.81)	-5.58 (-6.03, -5.14)	-	-
22	87.09 (86.79, 87.39)	83.27 (82.94, 83.6)	-3.82 (-4.27, -3.42)	71.35 (70.96, 71.75)	-15.20 (-16.24, -15.24)	81.54 (81.21, 81.87)	-5.55 (-6.00, -5.10)	-	-
23	87.13 (86.83, 87.43)	83.36 (83.03, 83.69)	-3.77 (-4.21, -3.37)	71.93 (71.54, 72.32)	-14.72 (-15.69, -14.70)	81.62 (81.29, 81.95)	-5.51 (-5.96, -5.07)	-	-
24	87.16 (86.86, 87.46)	83.43 (83.1, 83.76)	-3.73 (-4.18, -3.32)	72.45 (72.06, 72.84)	-14.07 (-15.21, -14.22)	81.7 (81.38, 82.03)	-5.46 (-5.91, -5.02)	-	-
25	87.21 (86.91, 87.51)	83.52 (83.19, 83.85)	-3.69 (-4.14, -3.29)	73.15 (72.76, 73.53)	-13.78 (-14.56, -13.58)	-	-	-	-
26	87.25 (86.95, 87.55)	83.6 (83.27, 83.93)	-3.65 (-4.10, -3.24)	73.47 (73.09, 73.86)	-13.65 (-14.26, -13.29)	-	-	-	-
27	87.29 (86.99, 87.59)	83.66 (83.33, 83.99)	-3.63 (-4.07, -3.21)	73.64 (73.26, 74.02)	-13.52 (-14.13, -13.16)	-	-	-	-

Months from end of school year	Pre-pandemic cohort	Pandemic cohorts							
	2018-19	2019-20		2020-21		2021-22		2022-23	
	Coverage, % (95% CI)	Coverage, % (95% CI)	Difference, % (95% CI)	Coverage, % (95% CI)	Difference, % (95% CI)	Coverage, % (95% CI)	Difference, % (95% CI)	Coverage, % (95% CI)	Difference, % (95% CI)
28	87.32 (87.02, 87.62)	83.72 (83.39, 84.05)	-3.60 (-4.05, -3.18)	73.8 (73.42, 74.18)	-13.47 (-14.01, -13.04)	-	-	-	-
29	87.37 (87.07, 87.67)	83.75 (83.43, 84.08)	-3.61 (-4.06, -3.16)	73.9 (73.52, 74.28)	-13.36 (-13.95, -12.98)	-	-	-	-
30	87.39 (87.10, 87.69)	83.83 (83.51, 84.16)	-3.56 (-4.00, -3.17)	74.04 (73.65, 74.42)	-13.26 (-13.84, -12.87)	-	-	-	-
31	87.42 (87.13, 87.72)	83.91 (83.59, 84.24)	-3.51 (-3.95, -3.12)	74.17 (73.78, 74.55)	-13.16 (-13.74, -12.78)	-	-	-	-
32	87.48 (87.18, 87.77)	84.06 (83.74, 84.39)	-3.41 (-3.85, -3.07)	74.32 (73.94, 74.7)	-13.09 (-13.64, -12.67)	-	-	-	-
33	87.52 (87.22, 87.82)	84.23 (83.9, 84.55)	-3.29 (-3.73, -2.97)	74.43 (74.05, 74.81)	-13.03 (-13.57, -12.61)	-	-	-	-
34	87.55 (87.25, 87.84)	84.41 (84.08, 84.73)	-3.14 (-3.58, -2.85)	74.52 (74.14, 74.9)	-13.01 (-13.51, -12.55)	-	-	-	-
35	87.63 (87.33, 87.92)	84.6 (84.28, 84.92)	-3.03 (-3.47, -2.70)	74.62 (74.24, 75.0)	-12.87 (-13.49, -12.53)	-	-	-	-
36	87.67 (87.37, 87.96)	84.75 (84.43, 85.07)	-2.92 (-3.36, -2.60)	74.79 (74.42, 75.17)	-12.87 (-13.35, -12.39)	-	-	-	-

Months from end of school year	Pre-pandemic cohort	Pandemic cohorts							
	2018-19	2019-20		2020-21		2021-22		2022-23	
	Coverage, % (95% CI)	Coverage, % (95% CI)	Difference, % (95% CI)	Coverage, % (95% CI)	Difference, % (95% CI)	Coverage, % (95% CI)	Difference, % (95% CI)	Coverage, % (95% CI)	Difference, % (95% CI)
37	87.72 (87.43, 88.02)	84.86 (84.54, 85.17)	-2.87 (-3.30, -2.49)	-	-	-	-	-	-
38	87.76 (87.47, 88.06)	84.9 (84.58, 85.22)	-2.86 (-3.30, -2.43)	-	-	-	-	-	-
39	87.80 (87.50, 88.09)	84.97 (84.65, 85.28)	-2.83 (-3.26, -2.43)	-	-	-	-	-	-
40	87.82 (87.53, 88.11)	85.02 (84.71, 85.34)	-2.79 (-3.23, -2.40)	-	-	-	-	-	-
41	87.85 (87.56, 88.15)	85.07 (84.75, 85.39)	-2.78 (-3.22, -2.36)	-	-	-	-	-	-
42	87.90 (87.61, 88.19)	85.11 (84.8, 85.43)	-2.79 (-3.22, -2.35)	-	-	-	-	-	-
43	87.94 (87.65, 88.24)	85.16 (84.85, 85.48)	-2.78 (-3.21, -2.36)	-	-	-	-	-	-
44	87.98 (87.69, 88.28)	85.22 (84.91, 85.54)	-2.76 (-3.19, -2.35)	-	-	-	-	-	-
45	88.04 (87.75, 88.33)	85.29 (84.97, 85.6)	-2.75 (-3.18, -2.33)	-	-	-	-	-	-

Months from end of school year	Pre-pandemic cohort	Pandemic cohorts							
	2018-19	2019-20		2020-21		2021-22		2022-23	
	Coverage, % (95% CI)	Coverage, % (95% CI)	Difference, % (95% CI)	Coverage, % (95% CI)	Difference, % (95% CI)	Coverage, % (95% CI)	Difference, % (95% CI)	Coverage, % (95% CI)	Difference, % (95% CI)
46	88.08 (87.79, 88.37)	85.33 (85.01, 85.64)	-2.76 (-3.19, -2.32)	-	-	-	-	-	-
47	88.14 (87.85, 88.44)	85.39 (85.08, 85.7)	-2.75 (-3.18, -2.33)	-	-	-	-	-	-
48	88.18 (87.89, 88.47)	85.46 (85.15, 85.77)	-2.72 (-3.15, -2.30)	-	-	-	-	-	-
49	88.21 (87.92, 88.50)	-	-	-	-	-	-	-	-
50	88.27 (87.98, 88.56)	-	-	-	-	-	-	-	-
51	88.31 (88.02, 88.60)	-	-	-	-	-	-	-	-
52	88.34 (88.06, 88.63)	-	-	-	-	-	-	-	-
53	88.37 (88.08, 88.66)	-	-	-	-	-	-	-	-
54	88.40 (88.12, 88.69)	-	-	-	-	-	-	-	-

Months from end of school year	Pre-pandemic cohort	Pandemic cohorts							
	2018-19	2019-20		2020-21		2021-22		2022-23	
	Coverage, % (95% CI)	Coverage, % (95% CI)	Difference, % (95% CI)	Coverage, % (95% CI)	Difference, % (95% CI)	Coverage, % (95% CI)	Difference, % (95% CI)	Coverage, % (95% CI)	Difference, % (95% CI)
55	88.44 (88.16, 88.73)	-	-	-	-	-	-	-	-
56	88.49 (88.20, 88.78)	-	-	-	-	-	-	-	-
57	88.53 (88.24, 88.82)	-	-	-	-	-	-	-	-
58	88.57 (88.29, 88.86)	-	-	-	-	-	-	-	-
59	88.60 (88.31, 88.89)	-	-	-	-	-	-	-	-
60	88.64 (88.35, 88.93)	-	-	-	-	-	-	-	-

Table S6. Monthly cumulative coverage for DTap vaccine for all Grade 1 cohorts, and difference in monthly coverage between pre-pandemic and pandemic cohorts. Month zero represents start of grade year (July) relevant to each cohort.

Months from start of school year	Pre-pandemic cohort	Pandemic cohorts							
	2018-19	2019-20		2020-21		2021-22		2022-23	
	Coverage, % (95% CI)	Coverage, % (95% CI)	Difference, % (95% CI)	Coverage, % (95% CI)	Difference, % (95% CI)	Coverage, % (95% CI)	Difference, % (95% CI)	Coverage, % (95% CI)	Difference, % (95% CI)
0	74.32 (73.96, 74.69)	74.99 (74.63, 75.36)	0.67 (0.15, 1.19)	73.53 (73.16, 73.90)	-0.79 (-1.31, -0.27)	69.66 (69.26, 70.05)	-4.67 (-5.21, -4.12)	66.00 (65.61, 66.39)	-8.33 (-8.86, -7.79)
1	75.85 (75.49, 76.21)	75.67 (75.30, 76.03)	-0.18 (-0.69, 0.33)	73.83 (73.46, 74.20)	-2.01 (-2.53, -1.50)	70.04 (69.64, 70.43)	-5.81 (-6.34, -5.27)	66.45 (66.06, 66.84)	-9.40 (-9.93, -8.87)
2	77.63 (77.28, 77.98)	76.19 (75.83, 76.55)	-1.45 (-1.95, -0.94)	74.13 (73.76, 74.49)	-3.51 (-4.01, -3.00)	70.49 (70.10, 70.89)	-7.14 (-7.67, -6.62)	67.07 (66.68, 67.45)	-10.57 (-11.09, -10.05)
3	78.65 (78.31, 79.00)	76.74 (76.39, 77.10)	-1.91 (-2.41, -1.41)	74.58 (74.21, 74.94)	-4.08 (-4.58, -3.57)	70.82 (70.43, 71.21)	-7.83 (-8.36, -7.31)	67.49 (67.10, 67.87)	-11.16 (-11.68, -10.65)
4	80.30 (79.97, 80.63)	77.79 (77.43, 78.14)	-2.51 (-3.00, -2.03)	75.73 (75.37, 76.09)	-4.57 (-5.06, -4.08)	71.14 (70.75, 71.53)	-9.16 (-9.67, -8.64)	68.25 (67.87, 68.64)	-12.05 (-12.55, -11.54)
5	81.50 (81.17, 81.83)	79.37 (79.02, 79.71)	-2.13 (-2.61, -1.66)	76.66 (76.31, 77.02)	-4.84 (-5.32, -4.36)	72.01 (71.62, 72.40)	-9.49 (-9.99, -8.98)	70.07 (69.69, 70.45)	-11.43 (-11.93, -10.93)
6	*	*	*	77.25 (76.90, 77.60)	*	74.95 (74.58, 75.32)	*	73.01 (72.64, 73.37)	*
7	*	*	*	*	*	76.87 (76.51, 77.23)	*	74.90 (74.54, 75.26)	*
8	*	*	*	*	*	78.90 (78.55, 79.25)	*	76.74 (76.39, 77.09)	*

Months from start of school year	Pre-pandemic cohort	Pandemic cohorts							
	2018-19	2019-20		2020-21		2021-22		2022-23	
	Coverage, % (95% CI)	Coverage, % (95% CI)	Difference, % (95% CI)	Coverage, % (95% CI)	Difference, % (95% CI)	Coverage, % (95% CI)	Difference, % (95% CI)	Coverage, % (95% CI)	Difference, % (95% CI)
9	*	*	*	*	*	*	*	77.92 (77.58, 78.26)	*
10	83.89 (83.58, 84.20)	79.43 (79.09, 79.77)	-4.46 (-4.92, -4.00)	77.47 (77.12, 77.82)	-6.41 (-6.88, -5.95)	79.87 (79.52, 80.21)	-4.02 (-4.48, -3.56)	78.04 (77.70, 78.38)	-5.85 (-6.31, -5.39)
11	84.14 (83.84, 84.45)	79.50 (79.16, 79.85)	-4.64 (-5.10, -4.18)	77.67 (77.32, 78.02)	-6.48 (-6.94, -6.01)	80.42 (80.08, 80.77)	-3.72 (-4.18, -3.26)	78.57 (78.23, 78.90)	-5.58 (-6.03, -5.12)
12	84.29 (83.99, 84.60)	79.65 (79.31, 79.99)	-4.64 (-5.10, -4.19)	77.81 (77.46, 78.16)	-6.48 (-6.95, -6.02)	80.63 (80.28, 80.97)	-3.67 (-4.13, -3.21)	78.81 (78.47, 79.14)	-5.49 (-5.94, -5.03)
13	84.49 (84.19, 84.79)	79.79 (79.45, 80.13)	-4.70 (-5.16, -4.24)	77.92 (77.57, 78.26)	-6.57 (-7.04, -6.11)	80.84 (80.50, 81.18)	-3.65 (-4.10, -3.19)	79.02 (78.68, 79.35)	-5.47 (-5.93, -5.02)
14	84.67 (84.37, 84.97)	80.05 (79.71, 80.39)	-4.62 (-5.08, -4.17)	78.04 (77.69, 78.39)	-6.63 (-7.09, -6.17)	81.04 (80.70, 81.38)	-3.63 (-4.08, -3.17)	79.29 (78.96, 79.63)	-5.38 (-5.83, -4.93)
15	84.84 (84.54, 85.14)	80.36 (80.03, 80.70)	-4.48 (-4.93, -4.03)	78.13 (77.78, 78.48)	-6.71 (-7.17, -6.25)	81.19 (80.85, 81.53)	-3.65 (-4.11, -3.20)	79.44 (79.11, 79.77)	-5.40 (-5.85, -4.95)
16	85.10 (84.80, 85.40)	80.50 (80.17, 80.84)	-4.60 (-5.05, -4.15)	78.20 (77.86, 78.55)	-6.90 (-7.35, -6.44)	81.34 (81.01, 81.68)	-3.76 (-4.21, -3.31)	79.89 (79.56, 80.22)	-5.21 (-5.66, -4.77)
17	85.43 (85.13, 85.73)	80.67 (80.33, 81.00)	-4.77 (-5.21, -4.32)	78.42 (78.07, 78.76)	-7.01 (-7.47, -6.56)	81.72 (81.39, 82.05)	-3.71 (-4.16, -3.27)	80.14 (79.81, 80.46)	-5.29 (-5.74, -4.85)
18	85.61 (85.31, 85.90)	80.80 (80.47, 81.14)	-4.80 (-5.25, -4.36)	78.92 (78.58, 79.26)	-6.69 (-7.14, -6.24)	82.26 (81.93, 82.59)	-3.34 (-3.79, -2.90)	80.40 (80.07, 80.72)	-5.21 (-5.65, -4.77)

Months from start of school year	Pre-pandemic cohort	Pandemic cohorts							
	2018-19	2019-20		2020-21		2021-22		2022-23	
	Coverage, % (95% CI)	Coverage, % (95% CI)	Difference, % (95% CI)	Coverage, % (95% CI)	Difference, % (95% CI)	Coverage, % (95% CI)	Difference, % (95% CI)	Coverage, % (95% CI)	Difference, % (95% CI)
19	85.63 (85.34, 85.93)	80.86 (80.53, 81.20)	-4.77 (-5.22, -4.32)	79.19 (78.85, 79.53)	-6.44 (-6.89, -5.99)	82.64 (82.32, 82.97)	-2.99 (-3.43, -2.55)	80.64 (80.32, 80.97)	-4.99 (-5.43, -4.55)
20	85.66 (85.37, 85.96)	80.93 (80.59, 81.26)	-4.74 (-5.18, -4.29)	79.50 (79.17, 79.84)	-6.16 (-6.61, -5.71)	83.05 (82.72, 83.37)	-2.61 (-3.05, -2.18)	80.86 (80.54, 81.19)	-4.80 (-5.23, -4.36)
21	85.68 (85.39, 85.98)	81.01 (80.68, 81.34)	-4.67 (-5.12, -4.23)	79.70 (79.36, 80.04)	-5.99 (-6.43, -5.54)	83.28 (82.95, 83.60)	-2.41 (-2.85, -1.97)	81.00 (80.68, 81.32)	-4.68 (-5.12, -4.25)
22	85.72 (85.42, 86.01)	81.06 (80.73, 81.39)	-4.66 (-5.10, -4.21)	79.76 (79.43, 80.10)	-5.95 (-6.40, -5.51)	83.34 (83.02, 83.66)	-2.38 (-2.81, -1.94)	81.08 (80.76, 81.40)	-4.64 (-5.08, -4.20)
23	85.74 (85.44, 86.03)	81.12 (80.78, 81.45)	-4.62 (-5.07, -4.18)	79.86 (79.52, 80.19)	-5.88 (-6.33, -5.44)	83.45 (83.13, 83.78)	-2.28 (-2.72, -1.85)	-	-
24	85.77 (85.48, 86.07)	81.16 (80.83, 81.49)	-4.62 (-5.06, -4.17)	79.90 (79.56, 80.23)	-5.88 (-6.32, -5.43)	83.53 (83.21, 83.85)	-2.25 (-2.68, -1.81)	-	-
25	85.80 (85.51, 86.09)	81.25 (80.91, 81.58)	-4.55 (-5.00, -4.11)	79.95 (79.62, 80.29)	-5.85 (-6.29, -5.40)	83.74 (83.42, 84.06)	-2.06 (-2.49, -1.63)	-	-
26	85.86 (85.57, 86.15)	81.39 (81.06, 81.72)	-4.47 (-4.91, -4.03)	80.02 (79.69, 80.36)	-5.84 (-6.28, -5.39)	84.05 (83.73, 84.36)	-1.82 (-2.25, -1.38)	-	-
27	85.91 (85.62, 86.20)	81.46 (81.13, 81.79)	-4.45 (-4.89, -4.01)	80.08 (79.75, 80.42)	-5.83 (-6.28, -5.39)	84.19 (83.88, 84.51)	-1.72 (-2.15, -1.29)	-	-
28	85.94 (85.64, 86.23)	81.49 (81.16, 81.82)	-4.45 (-4.89, -4.01)	80.23 (79.90, 80.57)	-5.70 (-6.15, -5.26)	84.97 (84.66, 85.28)	-0.97 (-1.39, -0.54)	-	-

Months from start of school year	Pre-pandemic cohort	Pandemic cohorts							
	2018-19	2019-20		2020-21		2021-22		2022-23	
	Coverage, % (95% CI)	Coverage, % (95% CI)	Difference, % (95% CI)	Coverage, % (95% CI)	Difference, % (95% CI)	Coverage, % (95% CI)	Difference, % (95% CI)	Coverage, % (95% CI)	Difference, % (95% CI)
29	85.97 (85.67, 86.26)	81.56 (81.23, 81.89)	-4.41 (-4.85, -3.97)	80.88 (80.55, 81.21)	-5.08 (-5.52, -4.64)	85.11 (84.80, 85.41)	-0.86 (-1.28, -0.44)	-	-
30	86.00 (85.70, 86.29)	81.83 (81.50, 82.16)	-4.17 (-4.60, -3.73)	81.90 (81.58, 82.22)	-4.09 (-4.53, -3.66)	85.24 (84.93, 85.55)	-0.75 (-1.18, -0.33)	-	-
31	86.01 (85.72, 86.30)	81.94 (81.62, 82.27)	-4.06 (-4.50, -3.63)	82.45 (82.14, 82.77)	-3.55 (-3.99, -3.12)	85.35 (85.05, 85.66)	-0.66 (-1.08, -0.23)	-	-
32	86.03 (85.74, 86.32)	82.12 (81.79, 82.44)	-3.91 (-4.35, -3.48)	82.98 (82.66, 83.29)	-3.05 (-3.48, -2.62)	85.50 (85.20, 85.81)	-0.52 (-0.94, -0.10)	-	-
33	86.05 (85.76, 86.34)	82.24 (81.92, 82.57)	-3.80 (-4.24, -3.37)	83.26 (82.95, 83.57)	-2.79 (-3.22, -2.36)	85.56 (85.26, 85.87)	-0.48 (-0.90, -0.06)	-	-
34	86.07 (85.78, 86.36)	82.28 (81.96, 82.61)	-3.79 (-4.22, -3.35)	83.32 (83.01, 83.63)	-2.75 (-3.18, -2.32)	85.61 (85.31, 85.91)	-0.46 (-0.88, -0.04)	-	-
35	86.08 (85.79, 86.37)	82.35 (82.03, 82.68)	-3.73 (-4.16, -3.29)	83.39 (83.08, 83.70)	-2.69 (-3.12, -2.26)	-	-	-	-
36	86.10 (85.81, 86.39)	82.38 (82.06, 82.71)	-3.71 (-4.15, -3.28)	83.47 (83.16, 83.79)	-2.62 (-3.05, -2.20)	-	-	-	-
37	86.12 (85.83, 86.41)	82.44 (82.12, 82.76)	-3.68 (-4.12, -3.25)	83.72 (83.41, 84.03)	-2.41 (-2.83, -1.98)	-	-	-	-
38	86.16 (85.87, 86.45)	82.49 (82.17, 82.81)	-3.67 (-4.10, -3.24)	83.98 (83.67, 84.28)	-2.19 (-2.61, -1.76)	-	-	-	-

Months from start of school year	Pre-pandemic cohort	Pandemic cohorts							
	2018-19	2019-20		2020-21		2021-22		2022-23	
	Coverage, % (95% CI)	Coverage, % (95% CI)	Difference, % (95% CI)	Coverage, % (95% CI)	Difference, % (95% CI)	Coverage, % (95% CI)	Difference, % (95% CI)	Coverage, % (95% CI)	Difference, % (95% CI)
39	86.19 (85.90, 86.48)	82.52 (82.20, 82.85)	-3.66 (-4.10, -3.23)	84.14 (83.83, 84.45)	-2.05 (-2.47, -1.63)	-	-	-	-
40	86.20 (85.91, 86.49)	82.56 (82.24, 82.88)	-3.64 (-4.07, -3.21)	85.25 (84.95, 85.55)	-0.95 (-1.36, -0.53)	-	-	-	-
41	86.22 (85.93, 86.51)	82.79 (82.47, 83.11)	-3.42 (-3.86, -2.99)	85.43 (85.13, 85.72)	-0.79 (-1.20, -0.37)	-	-	-	-
42	86.29 (86.00, 86.58)	83.17 (82.86, 83.49)	-3.12 (-3.55, -2.69)	85.55 (85.26, 85.85)	-0.74 (-1.15, -0.33)	-	-	-	-
43	86.34 (86.05, 86.63)	83.40 (83.08, 83.71)	-2.94 (-3.37, -2.51)	85.63 (85.33, 85.92)	-0.71 (-1.12, -0.30)	-	-	-	-
44	86.42 (86.13, 86.71)	83.66 (83.35, 83.98)	-2.76 (-3.19, -2.33)	85.72 (85.43, 86.01)	-0.70 (-1.11, -0.29)	-	-	-	-
45	86.47 (86.18, 86.76)	83.80 (83.49, 84.12)	-2.67 (-3.09, -2.24)	85.80 (85.50, 86.09)	-0.67 (-1.08, -0.26)	-	-	-	-
46	86.49 (86.20, 86.78)	83.83 (83.52, 84.15)	-2.66 (-3.08, -2.23)	85.83 (85.54, 86.12)	-0.66 (-1.07, -0.25)	-	-	-	-
47	86.51 (86.22, 86.80)	83.92 (83.61, 84.23)	-2.59 (-3.01, -2.17)	-	-	-	-	-	-
48	86.52 (86.24, 86.81)	83.95 (83.64, 84.27)	-2.57 (-2.99, -2.15)	-	-	-	-	-	-

Months from start of school year	Pre-pandemic cohort	Pandemic cohorts							
	2018-19	2019-20		2020-21		2021-22		2022-23	
	Coverage, % (95% CI)	Coverage, % (95% CI)	Difference, % (95% CI)	Coverage, % (95% CI)	Difference, % (95% CI)	Coverage, % (95% CI)	Difference, % (95% CI)	Coverage, % (95% CI)	Difference, % (95% CI)
49	86.54 (86.26, 86.83)	84.16 (83.85, 84.46)	-2.39 (-2.81, -1.97)	-	-	-	-	-	-
50	86.58 (86.30, 86.87)	84.42 (84.11, 84.73)	-2.16 (-2.58, -1.74)	-	-	-	-	-	-
51	86.59 (86.30, 86.88)	84.60 (84.29, 84.90)	-1.99 (-2.41, -1.57)	-	-	-	-	-	-
52	86.62 (86.33, 86.90)	86.09 (85.79, 86.38)	-0.53 (-0.94, -0.12)	-	-	-	-	-	-
53	86.69 (86.41, 86.98)	86.19 (85.90, 86.48)	-0.50 (-0.91, -0.09)	-	-	-	-	-	-
54	86.80 (86.52, 87.09)	86.26 (85.97, 86.56)	-0.54 (-0.95, -0.13)	-	-	-	-	-	-
55	86.89 (86.61, 87.17)	86.36 (86.07, 86.65)	-0.53 (-0.93, -0.12)	-	-	-	-	-	-
56	87.01 (86.72, 87.29)	86.45 (86.16, 86.74)	-0.56 (-0.96, -0.15)	-	-	-	-	-	-
57	87.06 (86.78, 87.34)	86.52 (86.23, 86.81)	-0.54 (-0.94, -0.13)	-	-	-	-	-	-
58	87.08 (86.80, 87.36)	86.56 (86.27, 86.85)	-0.51 (-0.92, -0.11)	-	-	-	-	-	-

Months from start of school year	Pre-pandemic cohort	Pandemic cohorts							
	2018-19	2019-20		2020-21		2021-22		2022-23	
	Coverage, % (95% CI)	Coverage, % (95% CI)	Difference, % (95% CI)	Coverage, % (95% CI)	Difference, % (95% CI)	Coverage, % (95% CI)	Difference, % (95% CI)	Coverage, % (95% CI)	Difference, % (95% CI)
59	87.10 (86.82, 87.38)	-	-	-	-	-	-	-	-
60	87.19 (86.91, 87.47)	-	-	-	-	-	-	-	-
61	87.74 (87.47, 88.02)	-	-	-	-	-	-	-	-
62	88.33 (88.06, 88.60)	-	-	-	-	-	-	-	-
63	88.49 (88.22, 88.75)	-	-	-	-	-	-	-	-
64	90.68 (90.44, 90.92)	-	-	-	-	-	-	-	-
65	90.71 (90.46, 90.95)	-	-	-	-	-	-	-	-
66	90.75 (90.51, 91.00)	-	-	-	-	-	-	-	-
67	90.85 (90.61, 91.09)	-	-	-	-	-	-	-	-
68	90.99 (90.75, 91.23)	-	-	-	-	-	-	-	-

Months from start of school year	Pre-pandemic cohort	Pandemic cohorts							
	2018-19	2019-20		2020-21		2021-22		2022-23	
	Coverage, % (95% CI)	Coverage, % (95% CI)	Difference, % (95% CI)	Coverage, % (95% CI)	Difference, % (95% CI)	Coverage, % (95% CI)	Difference, % (95% CI)	Coverage, % (95% CI)	Difference, % (95% CI)
69	91.07 (90.83, 91.31)	-	-	-	-	-	-	-	-
70	91.08 (90.84, 91.32)	-	-	-	-	-	-	-	-

*Data not available

Table S7. Monthly cumulative coverage for MMR/MMRV vaccine for all Grade 1 cohorts, and difference in monthly coverage between pre-pandemic and pandemic cohorts. Month zero represents start of grade year (September) relevant to each cohort.

Months from start of school year	Pre-pandemic cohort	Pandemic cohorts							
	2018-19	2019-20		2020-21		2021-22		2022-23	
	Coverage, % (95% CI)	Coverage, % (95% CI)	Difference, % (95% CI)	Coverage, % (95% CI)	Difference, % (95% CI)	Coverage, % (95% CI)	Difference, % (95% CI)	Coverage, % (95% CI)	Difference, % (95% CI)
0	76.62 (76.27, 76.98)	76.76 (76.41, 77.12)	0.14 (-0.36, 0.65)	74.96 (74.59, 75.32)	-1.67 (-2.17, -1.16)	71.07 (70.67, 71.46)	-5.56 (-6.08, -5.03)	67.22 (66.83, 67.60)	-9.40 (-9.93, -8.88)
1	78.06 (77.71, 78.40)	77.42 (77.07, 77.78)	-0.64 (-1.13, -0.14)	75.25 (74.88, 75.61)	-2.81 (-3.31, -2.31)	71.43 (71.04, 71.82)	-6.62 (-7.14, -6.10)	67.65 (67.27, 68.04)	-10.40 (-10.92, -9.89)
2	79.75 (79.42, 80.09)	77.95 (77.60, 78.30)	-1.80 (-2.29, -1.31)	75.53 (75.17, 75.89)	-4.22 (-4.72, -3.73)	71.88 (71.49, 72.27)	-7.87 (-8.39, -7.36)	68.24 (67.85, 68.62)	-11.52 (-12.03, 11.01)
3	80.68 (80.34, 81.01)	78.50 (78.15, 78.84)	-2.18 (-2.66, -1.70)	75.96 (75.60, 76.32)	-4.71 (-5.20, -4.22)	72.18 (71.80, 72.57)	-8.49 (-9.00, -7.98)	68.64 (68.26, 69.02)	-12.04 (-12.54, -11.53)
4	82.26 (81.94, 82.58)	79.46 (79.12, 79.81)	-2.80 (-3.27, -2.33)	77.06 (76.70, 77.41)	-5.21 (-5.68, -4.73)	72.49 (72.10, 72.87)	-9.78 (-10.28, -9.27)	69.36 (68.98, 69.74)	-12.91 (-13.40, -12.41)
5	83.42 (83.10, 83.73)	80.90 (80.57, 81.24)	-2.51 (-2.97, -2.06)	77.95 (77.60, 78.29)	-5.47 (-5.94, -5.00)	73.29 (72.91, 73.67)	-10.13 (-10.62, -9.63)	71.07 (70.70, 71.44)	-12.35 (-12.84, -11.86)
6	84.81 (84.51, 85.11)	81.69 (81.36, 82.02)	-3.12 (-3.56, -2.67)	78.51 (78.17, 78.86)	-6.29 (-6.75, -5.84)	76.00 (75.63, 76.37)	-8.81 (-9.28, -8.33)	73.83 (73.46, 74.19)	-10.98 (-11.45, -10.51)
7	85.88 (85.59, 86.17)	81.81 (81.48, 82.14)	-4.07 (-4.51, -3.63)	78.85 (78.50, 79.19)	-7.03 (-7.48, -6.58)	77.80 (77.44, 78.16)	-8.08 (-8.54, -7.62)	75.61 (75.26, 75.97)	-10.26 (-10.72, -9.81)
8	86.84 (86.55, 87.12)	81.90 (81.58, 82.23)	-4.93 (-5.37, -4.50)	79.07 (78.72, 79.41)	-7.77 (-8.22, -7.33)	79.69 (79.34, 80.04)	-7.15 (-7.59, -6.70)	77.36 (77.02, 77.71)	-9.47 (-9.92, -9.03)

Months from start of school year	Pre-pandemic cohort	Pandemic cohorts							
	2018-19	2019-20		2020-21		2021-22		2022-23	
	Coverage, % (95% CI)	Coverage, % (95% CI)	Difference, % (95% CI)	Coverage, % (95% CI)	Difference, % (95% CI)	Coverage, % (95% CI)	Difference, % (95% CI)	Coverage, % (95% CI)	Difference, % (95% CI)
9	87.48 (87.20, 87.76)	82.03 (81.70, 82.35)	-5.45 (-5.88, -5.03)	79.40 (79.06, 79.74)	-8.08 (-8.52, -7.64)	80.75 (80.41, 81.09)	-6.73 (-7.17, -6.29)	78.56 (78.23, 78.90)	-8.92 (-9.35, -8.48)
10	87.80 (87.53, 88.08)	82.13 (81.81, 82.46)	-5.67 (-6.10, -5.24)	79.59 (79.26, 79.93)	-8.21 (-8.65, -7.77)	81.16 (80.82, 81.49)	-6.65 (-7.08, -6.21)	78.92 (78.58, 79.25)	-8.89 (-9.32, -8.45)
11	88.06 (87.79, 88.33)	82.20 (81.87, 82.52)	-5.87 (-6.29, -5.44)	79.79 (79.46, 80.13)	-8.27 (-8.70, -7.83)	81.68 (81.34, 82.01)	-6.38 (-6.81, -5.95)	79.42 (79.08, 79.75)	-8.64 (-9.07, -8.21)
12	88.20 (87.92, 88.47)	82.33 (82.00, 82.65)	-5.87 (-6.29, -5.45)	79.92 (79.59, 80.26)	-8.27 (-8.70, -7.84)	81.88 (81.55, 82.22)	-6.31 (-6.74, -5.88)	79.65 (79.32, 79.98)	-8.55 (-8.97, -8.12)
13	88.38 (88.11, 88.65)	82.47 (82.15, 82.79)	-5.91 (-6.33, -5.49)	80.02 (79.68, 80.36)	-8.36 (-8.79, -7.93)	82.13 (81.79, 82.46)	-6.26 (-6.68, -5.83)	79.88 (79.55, 80.21)	-8.51 (-8.93, -8.08)
14	88.54 (88.27, 88.81)	82.70 (82.38, 83.02)	-5.84 (-6.26, -5.42)	80.14 (79.80, 80.47)	-8.40 (-8.83, -7.98)	82.34 (82.01, 82.67)	-6.20 (-6.62, -5.78)	80.18 (79.85, 80.50)	-8.37 (-8.79, -7.94)
15	88.68 (88.41, 88.95)	82.98 (82.66, 83.30)	-5.70 (-6.11, -5.28)	80.22 (79.89, 80.56)	-8.46 (-8.89, -8.03)	82.46 (82.13, 82.79)	-6.22 (-6.64, -5.80)	80.30 (79.97, 80.63)	-8.38 (-8.80, -7.96)
16	88.87 (88.61, 89.13)	83.10 (82.79, 83.42)	-5.77 (-6.18, -5.35)	80.30 (79.96, 80.63)	-8.57 (-9.00, -8.15)	82.61 (82.28, 82.94)	-6.26 (-6.68, -5.84)	80.46 (80.13, 80.78)	-8.41 (-8.83, -7.99)
17	89.12 (88.86, 89.38)	83.24 (82.92, 83.56)	-5.88 (-6.29, -5.47)	80.49 (80.15, 80.82)	-8.63 (-9.06, -8.21)	82.93 (82.61, 83.26)	-6.19 (-6.60, -5.77)	80.69 (80.37, 81.02)	-8.43 (-8.85, -8.01)
18	89.27 (89.01, 89.53)	83.37 (83.05, 83.68)	-5.90 (-6.31, -5.49)	80.92 (80.59, 81.25)	-8.34 (-8.76, -7.92)	83.43 (83.11, 83.75)	-5.83 (-6.25, -5.42)	80.94 (80.62, 81.26)	-8.33 (-8.74, -7.91)

Months from start of school year	Pre-pandemic cohort	Pandemic cohorts							
	2018-19	2019-20		2020-21		2021-22		2022-23	
	Coverage, % (95% CI)	Coverage, % (95% CI)	Difference, % (95% CI)	Coverage, % (95% CI)	Difference, % (95% CI)	Coverage, % (95% CI)	Difference, % (95% CI)	Coverage, % (95% CI)	Difference, % (95% CI)
19	89.29 (89.03, 89.55)	83.42 (83.11, 83.74)	-5.87 (-6.28, -5.46)	81.19 (80.86, 81.52)	-8.11 (-8.53, -7.69)	83.77 (83.45, 84.09)	-5.53 (-5.94, -5.11)	81.14 (80.81, 81.46)	-8.16 (-8.57, -7.74)
20	89.31 (89.05, 89.57)	83.48 (83.17, 83.80)	-5.82 (-6.23, -5.42)	81.48 (81.15, 81.80)	-7.83 (-8.25, -7.41)	84.12 (83.80, 84.43)	-5.19 (-5.60, -4.78)	81.33 (81.01, 81.65)	-7.98 (-8.39, -7.56)
21	89.34 (89.08, 89.60)	83.56 (83.25, 83.88)	-5.77 (-6.18, -5.37)	81.65 (81.33, 81.98)	-7.68 (-8.10, -7.27)	84.36 (84.05, 84.67)	-4.98 (-5.38, -4.57)	81.44 (81.12, 81.76)	-7.90 (-8.31, -7.49)
22	89.37 (89.11, 89.63)	83.61 (83.30, 83.92)	-5.76 (-6.17, -5.35)	81.71 (81.38, 82.03)	-7.66 (-8.08, -7.25)	84.42 (84.11, 84.73)	-4.95 (-5.36, -4.54)	81.50 (81.18, 81.82)	-7.87 (-8.28, -7.46)
23	89.40 (89.14, 89.65)	83.66 (83.35, 83.98)	-5.73 (-6.14, -5.32)	81.80 (81.47, 82.12)	-7.60 (-8.01, -7.18)	84.53 (84.21, 84.84)	-4.87 (-5.27, -4.46)	-	-
24	89.43 (89.17, 89.69)	83.71 (83.39, 84.02)	-5.72 (-6.13, -5.32)	81.83 (81.51, 82.16)	-7.60 (-8.01, -7.18)	84.60 (84.29, 84.91)	-4.83 (-5.24, -4.43)	-	-
25	89.44 (89.19, 89.70)	83.77 (83.46, 84.09)	-5.67 (-6.08, -5.26)	81.89 (81.57, 82.21)	-7.55 (-7.97, -7.14)	84.80 (84.49, 85.11)	-4.64 (-5.04, -4.24)	-	-
26	89.50 (89.24, 89.75)	83.91 (83.60, 84.23)	-5.58 (-5.99, -5.18)	81.93 (81.61, 82.26)	-7.56 (-7.98, -7.15)	85.12 (84.82, 85.43)	-4.37 (-4.77, -3.97)	-	-
27	89.56 (89.30, 89.82)	83.97 (83.66, 84.28)	-5.59 (-5.99, -5.18)	82.00 (81.68, 82.32)	-7.56 (-7.97, -7.15)	85.28 (84.97, 85.58)	-4.28 (-4.68, -3.88)	-	-
28	89.58 (89.33, 89.84)	84.01 (83.70, 84.32)	-5.58 (-5.98, -5.17)	82.13 (81.81, 82.45)	-7.45 (-7.86, -7.04)	85.45 (85.15, 85.76)	-4.13 (-4.53, -3.73)	-	-

Months from start of school year	Pre-pandemic cohort	Pandemic cohorts							
	2018-19	2019-20		2020-21		2021-22		2022-23	
	Coverage, % (95% CI)	Coverage, % (95% CI)	Difference, % (95% CI)	Coverage, % (95% CI)	Difference, % (95% CI)	Coverage, % (95% CI)	Difference, % (95% CI)	Coverage, % (95% CI)	Difference, % (95% CI)
29	89.61 (89.35, 89.87)	84.08 (83.77, 84.39)	-5.53 (-5.93, -5.13)	82.73 (82.41, 83.04)	-6.88 (-7.29, -6.48)	85.57 (85.27, 85.87)	-4.04 (-4.44, -3.64)	-	-
30	89.62 (89.37, 89.88)	84.31 (84.00, 84.62)	-5.31 (-5.71, -4.91)	83.65 (83.34, 83.96)	-5.98 (-6.38, -5.57)	85.69 (85.39, 86.00)	-3.93 (-4.33, -3.54)	-	-
31	89.63 (89.38, 89.89)	84.42 (84.12, 84.73)	-5.21 (-5.61, -4.81)	84.17 (83.86, 84.47)	-5.46 (-5.86, -5.06)	85.81 (85.50, 86.11)	-3.83 (-4.22, -3.43)	-	-
32	89.64 (89.39, 89.90)	84.58 (84.27, 84.88)	-5.07 (-5.47, -4.67)	84.63 (84.32, 84.93)	-5.02 (-5.41, -4.62)	85.93 (85.63, 86.23)	-3.71 (-4.10, -3.32)	-	-
33	89.65 (89.39, 89.90)	84.69 (84.39, 85.00)	-4.95 (-5.35, -4.56)	84.95 (84.65, 85.25)	-4.70 (-5.10, -4.31)	85.99 (85.69, 86.29)	-3.66 (-4.05, -3.26)	-	-
34	89.66 (89.41, 89.92)	84.73 (84.43, 85.04)	-4.93 (-5.33, -4.53)	85.00 (84.70, 85.30)	-4.66 (-5.06, -4.27)	86.03 (85.73, 86.33)	-3.63 (-4.02, -3.24)	-	-
35	89.67 (89.42, 89.93)	84.80 (84.50, 85.11)	-4.87 (-5.27, -4.47)	85.07 (84.77, 85.37)	-4.61 (-5.00, -4.21)	-	-	-	-
36	89.70 (89.44, 89.95)	84.83 (84.52, 85.13)	-4.87 (-5.27, -4.47)	85.14 (84.84, 85.44)	-4.56 (-4.95, -4.16)	-	-	-	-
37	89.74 (89.48, 89.99)	84.87 (84.57, 85.17)	-4.86 (-5.26, -4.47)	85.40 (85.10, 85.70)	-4.34 (-4.73, -3.94)	-	-	-	-
38	89.77 (89.52, 90.03)	84.93 (84.62, 85.23)	-4.85 (-5.24, -4.45)	85.66 (85.37, 85.96)	-4.11 (-4.50, -3.72)	-	-	-	-

Months from start of school year	Pre-pandemic cohort	Pandemic cohorts							
	2018-19	2019-20		2020-21		2021-22		2022-23	
	Coverage, % (95% CI)	Coverage, % (95% CI)	Difference, % (95% CI)	Coverage, % (95% CI)	Difference, % (95% CI)	Coverage, % (95% CI)	Difference, % (95% CI)	Coverage, % (95% CI)	Difference, % (95% CI)
39	89.80 (89.54, 90.05)	84.96 (84.66, 85.27)	-4.83 (-5.23, -4.44)	85.81 (85.52, 86.10)	-3.99 (-4.37, -3.60)	-	-	-	-
40	89.80 (89.55, 90.06)	85.00 (84.70, 85.30)	-4.80 (-5.20, -4.41)	85.94 (85.65, 86.23)	-3.87 (-4.25, -3.48)	-	-	-	-
41	89.82 (89.57, 90.08)	85.19 (84.89, 85.49)	-4.63 (-5.02, -4.24)	86.10 (85.81, 86.39)	-3.72 (-4.10, -3.33)	-	-	-	-
42	89.88 (89.63, 90.14)	85.51 (85.21, 85.81)	-4.37 (-4.76, -3.98)	86.23 (85.94, 86.52)	-3.66 (-4.04, -3.27)	-	-	-	-
43	89.93 (89.67, 90.18)	85.70 (85.40, 86.00)	-4.23 (-4.62, -3.84)	86.31 (86.02, 86.60)	-3.61 (-4.00, -3.23)	-	-	-	-
44	89.98 (89.73, 90.23)	85.93 (85.64, 86.23)	-4.05 (-4.44, -3.66)	86.40 (86.11, 86.69)	-3.58 (-3.96, -3.20)	-	-	-	-
45	90.02 (89.77, 90.27)	86.08 (85.79, 86.37)	-3.94 (-4.33, -3.55)	86.46 (86.17, 86.75)	-3.56 (-3.94, -3.18)	-	-	-	-
46	90.04 (89.78, 90.29)	86.11 (85.81, 86.40)	-3.93 (-4.32, -3.54)	86.49 (86.21, 86.78)	-3.54 (-3.92, -3.16)	-	-	-	-
47	90.05 (89.80, 90.30)	86.19 (85.90, 86.48)	-3.86 (-4.25, -3.47)	-	-	-	-	-	-
48	90.06 (89.81, 90.31)	86.23 (85.94, 86.52)	-3.83 (-4.21, -3.44)	-	-	-	-	-	-

Months from start of school year	Pre-pandemic cohort	Pandemic cohorts							
	2018-19	2019-20		2020-21		2021-22		2022-23	
	Coverage, % (95% CI)	Coverage, % (95% CI)	Difference, % (95% CI)	Coverage, % (95% CI)	Difference, % (95% CI)	Coverage, % (95% CI)	Difference, % (95% CI)	Coverage, % (95% CI)	Difference, % (95% CI)
49	90.07 (89.82, 90.32)	86.41 (86.12, 86.71)	-3.66 (-4.04, -3.27)	-	-	-	-	-	-
50	90.10 (89.84, 90.35)	86.69 (86.41, 86.98)	-3.40 (-3.78, -3.02)	-	-	-	-	-	-
51	90.11 (89.86, 90.36)	86.86 (86.57, 87.14)	-3.25 (-3.63, -2.87)	-	-	-	-	-	-
52	90.13 (89.88, 90.38)	86.98 (86.70, 87.27)	-3.15 (-3.53, -2.77)	-	-	-	-	-	-
53	90.19 (89.94, 90.44)	87.08 (86.80, 87.37)	-3.11 (-3.49, -2.73)	-	-	-	-	-	-
54	90.27 (90.02, 90.52)	87.14 (86.86, 87.43)	-3.13 (-3.50, -2.75)	-	-	-	-	-	-
55	90.33 (90.08, 90.58)	87.25 (86.96, 87.53)	-3.08 (-3.46, -2.71)	-	-	-	-	-	-
56	90.43 (90.18, 90.67)	87.34 (87.05, 87.62)	-3.09 (-3.47, -2.72)	-	-	-	-	-	-
57	90.47 (90.23, 90.72)	87.39 (87.11, 87.67)	-3.08 (-3.46, -2.71)	-	-	-	-	-	-
58	90.49 (90.24, 90.73)	87.42 (87.14, 87.70)	-3.07 (-3.44, -2.69)	-	-	-	-	-	-

Months from start of school year	Pre-pandemic cohort	Pandemic cohorts							
	2018-19	2019-20		2020-21		2021-22		2022-23	
	Coverage, % (95% CI)	Coverage, % (95% CI)	Difference, % (95% CI)	Coverage, % (95% CI)	Difference, % (95% CI)	Coverage, % (95% CI)	Difference, % (95% CI)	Coverage, % (95% CI)	Difference, % (95% CI)
59	90.50 (90.25, 90.75)	-	-	-	-	-	-	-	-
60	90.58 (90.33, 90.82)	-	-	-	-	-	-	-	-
61	91.05 (90.81, 91.29)	-	-	-	-	-	-	-	-
62	91.54 (91.31, 91.78)	-	-	-	-	-	-	-	-
63	91.68 (91.44, 91.91)	-	-	-	-	-	-	-	-
64	91.70 (91.47, 91.93)	-	-	-	-	-	-	-	-
65	91.74 (91.51, 91.97)	-	-	-	-	-	-	-	-
66	91.79 (91.56, 92.02)	-	-	-	-	-	-	-	-
67	91.92 (91.69, 92.15)	-	-	-	-	-	-	-	-
68	92.05 (91.82, 92.28)	-	-	-	-	-	-	-	-

Months from start of school year	Pre-pandemic cohort	Pandemic cohorts							
	2018-19	2019-20		2020-21		2021-22		2022-23	
	Coverage, % (95% CI)	Coverage, % (95% CI)	Difference, % (95% CI)	Coverage, % (95% CI)	Difference, % (95% CI)	Coverage, % (95% CI)	Difference, % (95% CI)	Coverage, % (95% CI)	Difference, % (95% CI)
69	92.13 (91.90, 92.35)	-	-	-	-	-	-	-	-
70	92.14 (91.92, 92.37)	-	-	-	-	-	-	-	-
